# Posterior Parietal Cortex Drives Inferotemporal Activations During Three-Dimensional Object Vision

**DOI:** 10.1371/journal.pbio.1002445

**Published:** 2016-04-15

**Authors:** Ilse C. Van Dromme, Elsie Premereur, Bram-Ernst Verhoef, Wim Vanduffel, Peter Janssen

**Affiliations:** 1 KU Leuven, Laboratorium voor Neuro- en Psychofysiologie, Leuven, Belgium; 2 Department of Neurobiology, University of Chicago, Chicago, Illinois, United States of America; 3 Harvard Medical School, Boston, Massachusetts, United States of America; 4 MGH Martinos Center for Biomedical Imaging, Charlestown, Massachusetts, United States of America; Oxford University, UNITED KINGDOM

## Abstract

The primate visual system consists of a ventral stream, specialized for object recognition, and a dorsal visual stream, which is crucial for spatial vision and actions. However, little is known about the interactions and information flow between these two streams. We investigated these interactions within the network processing three-dimensional (3D) object information, comprising both the dorsal and ventral stream. Reversible inactivation of the macaque caudal intraparietal area (CIP) during functional magnetic resonance imaging (fMRI) reduced fMRI activations in posterior parietal cortex in the dorsal stream and, surprisingly, also in the inferotemporal cortex (ITC) in the ventral visual stream. Moreover, CIP inactivation caused a perceptual deficit in a depth-structure categorization task. CIP-microstimulation during fMRI further suggests that CIP projects via posterior parietal areas to the ITC in the ventral stream. To our knowledge, these results provide the first causal evidence for the flow of visual 3D information from the dorsal stream to the ventral stream, and identify CIP as a key area for depth-structure processing. Thus, combining reversible inactivation and electrical microstimulation during fMRI provides a detailed view of the functional interactions between the two visual processing streams.

## Abstract 2

A study in which monkeys examined images of three-dimensional shapes, showing that reversible inactivation of a dorsal stream area affects functional activation in the ventral visual stream.

## Author Summary

Many different areas of the brain are utilized to analyze the three-dimensional shape of the objects we see and grasp (e.g., a sphere compared to a disk). In this study, we temporarily inactivated one of these areas in the monkey parietal cortex and measured the effect on the network of brain areas involved in three-dimensional shape processing using functional brain imaging. Surprisingly, reversible inactivation of a parietal area not only reduced the functional activations in other parietal areas, but also in distantly separate brain areas such as the inferotemporal cortex. Moreover, parietal inactivation also caused a significant perceptual deficit in a depth-structure discrimination task. Electrical microstimulation of the inactivation site revealed that almost all of the observed effects were indirect, i.e., arising through a different brain area. Thus, a combination of functional brain imaging, causal perturbation methods, and behavioral measurements in monkeys can elucidate the flow of three-dimensional object information in the primate brain.

## Introduction

The primate visual cortex consists of a large number of cortical areas that collaborate to compute neural representations of the external world. In both the dorsal and the ventral visual stream of the macaque monkey, these cortical networks are organized into distinct hierarchies that provide progressively more elaborate representations of specific stimulus features such as motion [1], color [2], and disparity [3,4]; stimulus categories such as faces [5] and bodies [6]; and actions such as reaching, grasping, and saccadic eye movements [7–11]. To understand the computations at each level within such a hierarchy, it is crucial to know the components of the network, the anatomical connectivity between these regions, and the properties of individual neurons. However, how visual information flows between the different components of the network is frequently unknown.

Understanding how visual information flows is particularly challenging when investigating the functional interactions between the dorsal and the ventral visual streams. Lesion studies have demonstrated that each visual processing stream is specialized [12,13] and can function—at least to some extent—independently of the other stream [14,15]. Both anatomical [16] and functional [17–19] evidence strongly suggest that the dorsal and ventral stream interact during object vision, but no study has been able to show conclusively which information is transferred between the two streams.

To compute the depth structure of objects (e.g., concave or convex), the visual system recruits cortical regions in both the ventral and the dorsal visual stream [20–23]. Here, we first charted the network of cortical areas implicated in three-dimensional (3D) object vision using fMRI in awake monkeys. We then reversibly inactivated the caudal intraparietal area (CIP) during fMRI and observed widespread but highly selective changes in fMRI activations in the depth structure network comprising both posterior parietal and inferotemporal cortex (ITC). Moreover, CIP inactivation caused a significant reduction in performance in a depth structure categorization task. Electrical microstimulation of CIP during fMRI mainly activated areas outside the 3D network, indicating that the effects of CIP inactivation may originate as an indirect effect through posterior parietal cortex. To our knowledge, these results provide the first evidence for a causal contribution of a dorsal stream area to fMRI activations in the ventral visual stream.

## Results

We performed fMRI (monkeys M, K, R, and S), reversible inactivation-fMRI (monkeys M, R, and S), electrical microstimulation-fMRI (EM-fMRI; monkeys M, R, and D), reversible inactivation-psychophysics (monkeys R and S), and single-cell recordings (monkeys R and D) in five male rhesus monkeys having excellent stereopsis [24]. Depth-structure sensitivity was defined as a significant activation for the contrast [CS–CC]–[FS–FC], where CS = curved stereo, CC = curved control, FS = flat stereo, and FC = flat control (*p* < 0.05, family wise error [fwe] corrected for multiple comparisons); i.e., voxels that were more activated by curved surfaces (compared to their control stimuli, which consisted of one of the monocular images presented to both eyes simultaneously) than by flat surfaces (compared to their control stimuli) presented at the same mean disparities. This contrast amounts to a significant interaction between the factors “curvature” (curved or flat) and “disparity” (present or absent). Note, however, that our curved stimuli differed from the flat stimuli by the presence of second-order disparity variations (as in concave and convex) and first-order disparity variations (as in planar tilted surfaces). Hence, in our design, the factor “curvature” compared higher-order disparity stimuli to zero-order disparity stimuli.

### The Network of Depth Structure-Sensitive Cortical Areas

To obtain an overview of the cortical network that is sensitive to depth structure, we scanned four monkeys in a block design (423 runs in total; Monkey M, 113 runs; Monkey R, 127 runs; Monkey S, 94 runs; and Monkey K, 89 runs) in 21 scanning sessions (Monkey M, 5; Monkey R, 6; Monkey S, 4; and Monkey K, 6). We calculated the interaction between the factors “curvature” and “disparity” on the group data (using 89 runs for each monkey), by computing the contrast [CS–CC]–[FS–FC], where CS = curved stereo, CC = curved control, FS = flat stereo and FC = flat control. As in previous studies [20,22,25], this contrast aims to identify regions that are activated more strongly by curved stimuli (compared to their control stimuli) than by flat stimuli (compared to their control stimuli) presented at different disparities.

**Fig 1:**
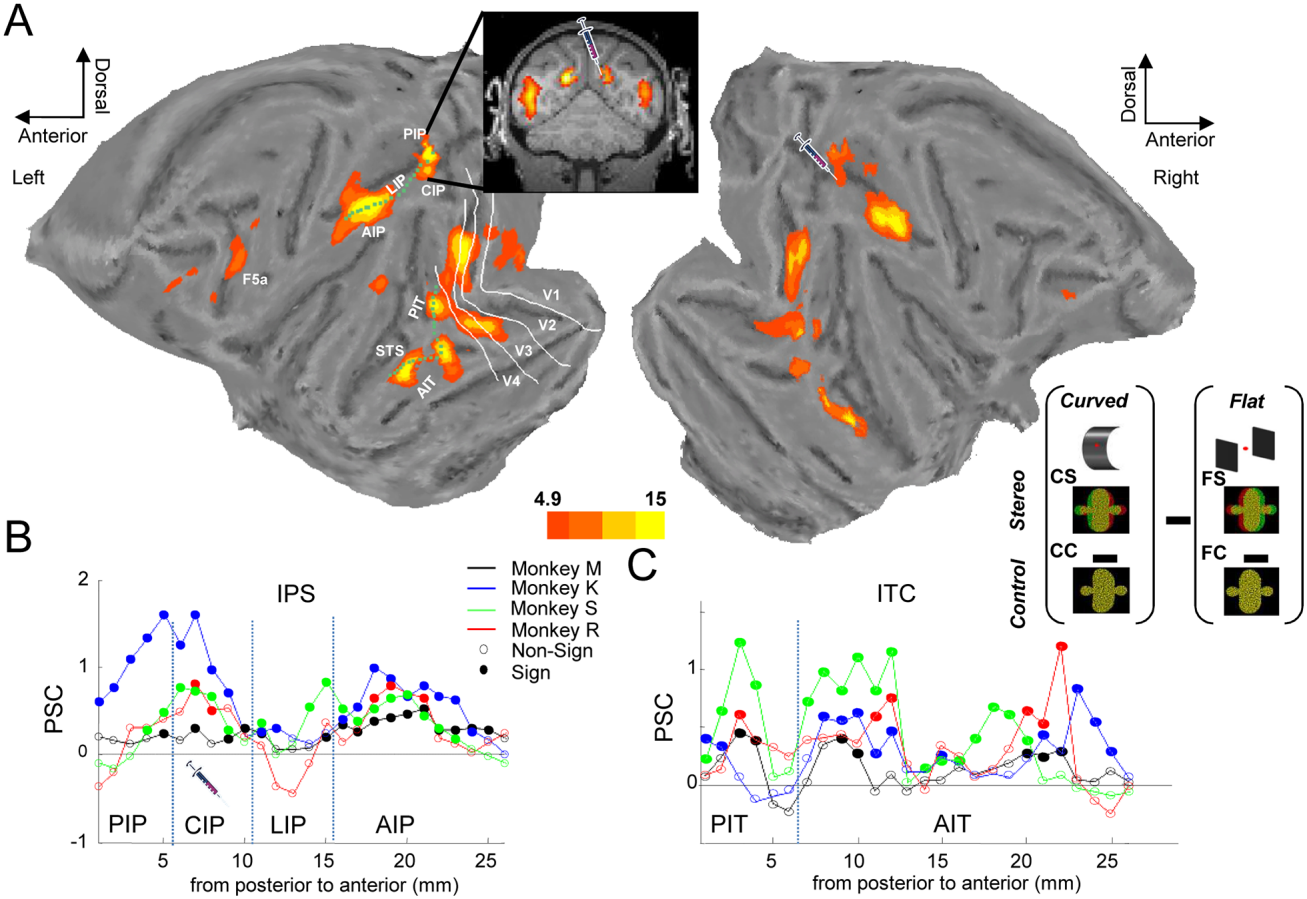
Overview of depth-structure related fMRI activations. A. Group average of the [(curved stereo–curved control)–(flat stereo–flat control)] interaction effect plotted on a flat map of the M12 anatomical template. The inset shows a coronal image illustrating depth-structure related activations in the caudal intraparietal sulcus (IPS). The two green dotted lines illustrate the paths drawn through the IPS and along the inferotemporal cortex (ITC), which were used to calculate the size of the interaction effect in individual animals. The white contours indicate the borders of the early visual areas V1–V4. The picture of the injection syringe indicates the area that was inactivated in the following experiments. *t* values in [27]: stereo.img/hdr. B. Percent signal change of the interaction effect calculated along a path drawn along the IPS of the left hemisphere in four monkeys (black: monkey M, red: monkey R, blue: monkey K, green: monkey S). The *x*-axis denotes distance in millimeters along the path, with zero corresponding to the medial bank of the caudal IPS (area PIP) and 25 corresponding to the anterior tip of the IPS. Filled symbols indicate significant (curvature x disparity interaction effect) activations, open symbols indicate nonsignificant activations. Raw values in monkey_path_ips in [27]. C. Percent signal change in the interaction effect calculated along a path drawn along the ITC in four monkeys. Zero corresponds to the depth-structure activation in V4/PIT, whereas 25 corresponds to the most rostral lower bank of the superior temporal sulcus (STS). Same conventions as in B. Raw values in monkey_path_itc in [27].

The network of cortical areas sensitive to depth structure was remarkably extensive (Fig 1A). In line with previous studies [17,20,25], the anterior lateral bank of the intraparietal sulcus (IPS) was more strongly activated by curved surfaces than by flat surfaces at different disparities. However, in contrast to previous investigations [20,22], we also observed additional activations in the caudal IPS region—comprising both the lateral and the medial bank of the caudal IPS. Based on the anatomical locations of depth-structure-related activations (see inset in Fig 1A), this caudal IPS region consisted of area CIP on the lateral bank and the posterior intraparietal area (PIP) on the medial bank of the IPS. For illustrative purposes, we calculated the percent signal change (PSC) of the curvature x disparity interaction effect along a path drawn through the IPS of the left hemisphere from caudomedially (area PIP) to rostrolaterally (anterior intraparietal area or AIP, Fig 1B), for each of the four monkeys separately. Note that highly similar results were obtained for both hemispheres. Fig 1B illustrates that in every animal, both the caudal and the anterior lateral IPS showed significant depth-structure-related activations. In occipital cortex, we measured significant activations related to depth structure in and around the lunate sulcus and the inferior occipital sulcus, corresponding to dorsal and ventral areas V3, V4, and parts of V1 and V2 [26].

Surprisingly, a prominent activation pattern related to depth structure sensitivity was also observed in ITC. Both hemispheres showed strong activations in posterior ITC (which partially corresponds to TEO [26]), on the shoulder and in the lower bank of the superior temporal sulcus (STS), and more anteriorly in anterior IT (AIT); posteriorly near the posterior middle temporal sulcus (PMTS) on the temporal convexity, and anteriorly on the shoulder and lower bank of the rostral STS, a region most likely corresponding to the recording area in previous single-cell studies [21,28]. We calculated the percent signal change along a path through temporal cortex running from posterior inferotemporal cortex (PIT) to AIT (Fig 1A). Each of the four subjects showed significant (although to varying degrees; compare monkey M to monkey S: one sample *t* tests, *p* < 0.05; Fig 1C) depth-structure-related activations in the ITC: a posterior patch corresponding to PIT, and more anterior activations in AIT (largely corresponding to area TE, Fig 1C). Additional, but smaller, activations related to depth structure sensitivity were located in the anterior subsector of ventral premotor cortex (area F5a, significant in three out of four monkeys [22,29]) and in the prefrontal cortex (area 46 and possibly area 45 in the left hemisphere).

### The Effect of Reversible Inactivation of CIP during fMRI

**Fig 2:**
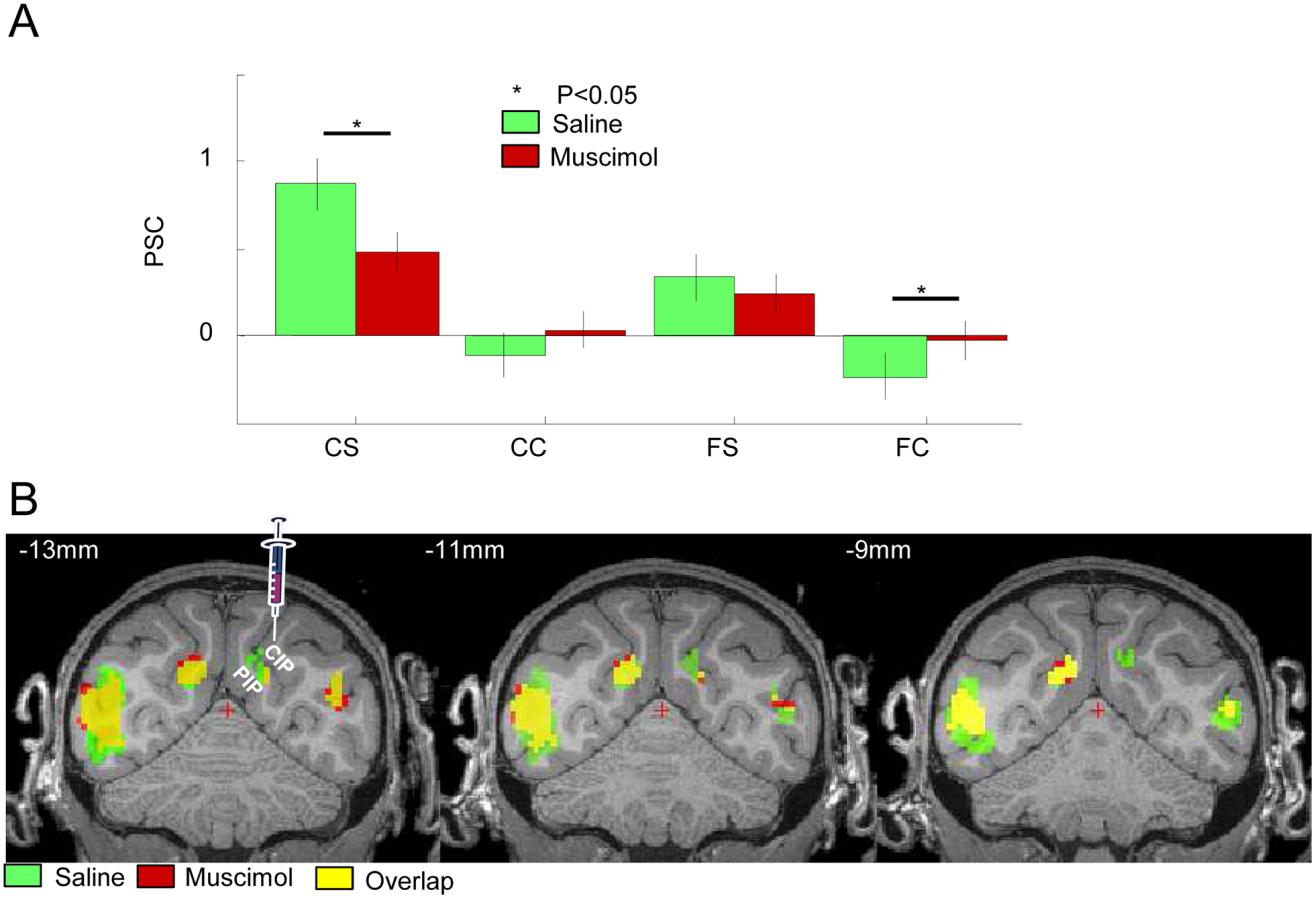
Effect of muscimol injection in CIP on the fMRI activations in the caudal IPS. A. Group average percent signal change (compared to fixation) in the four experimental conditions. CS: curved stereo, CC: curved control, FS: flat stereo, FC: flat control. Green bars indicate saline sessions; red bars indicate muscimol sessions. * = *p* < 0.05. Raw values in [27]: cIPSr. B. Group average of the contrast [(curved stereo–curved control)–(flat stereo–flat control)] in the caudal IPS during saline and muscimol sessions (at *p* < 0.05 FWE corrected for multiple comparisons), plotted on coronal sections of the M12 anatomical template (average of three monkeys). Green: saline sessions, red: muscimol sessions, yellow: overlap between saline and muscimol sessions. Green voxels indicate significant depth structure activations during saline sessions but not during muscimol sessions, hence voxels that were affected by CIP inactivation. Red voxels indicate significant activations during muscimol sessions but not during saline session (mainly in the contralateral hemisphere). The left panel indicates the approximate location of the muscimol injection (see also S1B Fig). *t* values in [27]: stereo_sal.img/hdr and stereo_mus.img/hdr.

To assess the role of area CIP in the network of cortical areas sensitive to depth structure, we reversibly inactivated CIP in one hemisphere while the animals were passively fixating curved and flat surfaces during fMRI. To verify that we had successfully inactivated CIP, we first investigated the effect of muscimol injections on the fMRI activations elicited by curved surfaces in the caudal IPS. Because it was difficult to dissociate the fMRI activations in the medial bank from those in the lateral bank of the caudal IPS, we evaluated the effect of muscimol in a single region of interest (ROI) that encompassed both PIP and CIP (but see S2A Fig for the effects on the medial and lateral bank of the caudal IPS). Muscimol injections significantly reduced (unpaired *t* test, T(304) = 2.434; *p* = 0.015) the fMRI activations evoked by curved surfaces in the caudal IPS of the inactivated hemisphere (Fig 2A). Analysis of Variance (ANOVA) on the PSC in the caudal IPS ROI with factors condition and inactivation (muscimol versus saline) showed that the main effect of condition was highly significant (F(3,1216) = 17.15; *p* < 0.001), but because muscimol caused fewer deactivations in the control conditions (curved control and flat control), the main effect of inactivation was not significant (F(1,1216) = 1.52; *p* = 0.22), while the interaction between the factors condition and inactivation almost reached significance (F(3,1216) = 2.36; *p* = 0.06). Saline injections did not produce any significant reduction in PSC, since the size of the curvature x disparity effect was comparable between saline sessions (average PSC over runs = 0.4) and the first fMRI experiment (PSC = 0.41; T = 0.3471, *p* = 0.73). We plotted the depth-structure interaction effect (curvature x disparity) under saline and muscimol conditions (at *p* < 0.05 FWE corrected for multiple comparisons) on coronal images of the MRI template (Fig 2B, group data, green voxels indicate depth structure activations during saline but not during muscimol sessions). Muscimol injections in the lateral bank of the caudal IPS reduced the depth-structure-related activations both in the medial and in the lateral bank of the caudal IPS (ROI defined on the basis of the saline injections; significant reduction of the curvature x disparity interaction effect in the PSC in the caudal IPS ROI, T = 1.98; *p* = 0.049). We also calculated the effect of muscimol injection on the depth-structure-related PSC within the caudal IPS region that was inactivated, which was estimated based on the anatomical MRI after the injection of the contrast agent Dotarem (S1B Fig). Restricting the analysis of the effect of muscimol injection to a ROI defined by the sum of all Dotarem ROIs of the three monkeys resulted in a significant effect of inactivation on the PSC of the main effect of disparity (T[304] = 2.8194; *p* = 0.005127). Finally, the path drawn along the IPS illustrated in S2A Fig also shows that CIP inactivation caused a reduction in PSC both in the lateral (CIP) and in the medial (PIP) bank of the caudal IPS. Note that Fig 2 also shows a very small number of red voxels in the inactivated hemisphere, indicating higher activations following CIP inactivation. These results are, however, most likely due to imperfect warping to the anatomical template.

**Fig 3:**
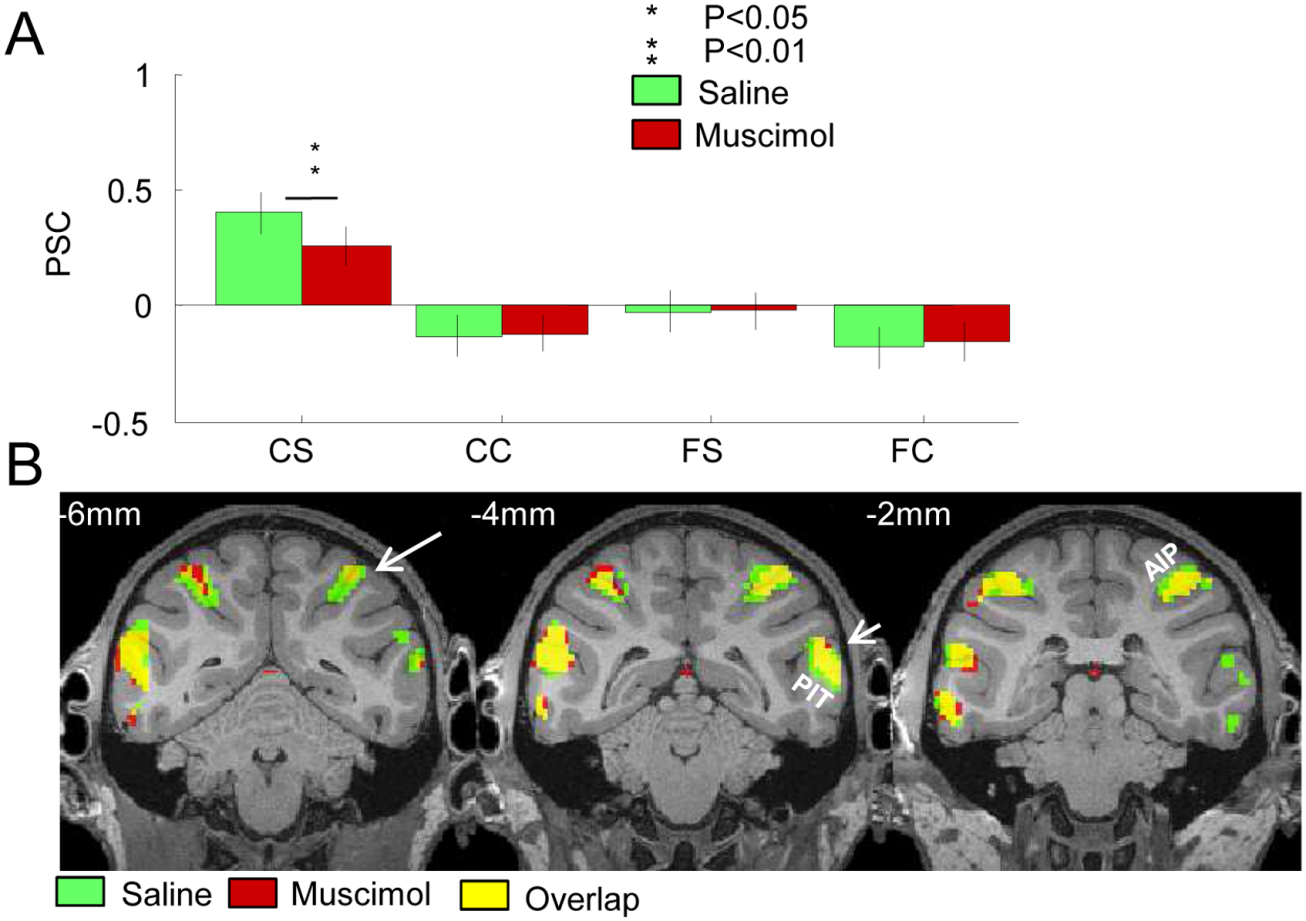
Effect of muscimol injection in CIP on the fMRI activations in the anterior IPS. A. Group average percent signal change (compared to fixation) in the four experimental conditions in the anterior IPS of the inactivated (right) hemisphere. Same conventions as in Fig 2A. * = *p* < 0.05. ** = *p* < 0.01, both corrected for multiple comparisons. Raw values in [27]: aIPSr. B. Group average of the curvature x stereo interaction effect [(curved stereo–curved control)–(flat stereo–flat control)] in the anterior IPS during saline and muscimol sessions, plotted on coronal sections of the M12 anatomical template (average of three monkeys; *p* < 0.05 FWE corrected). The arrow in the left panel indicates the significant reduction in depth-structure-related activations in the anterior IPS (yellow voxels indicate the overlap between saline and muscimol sessions). The middle panel also illustrates the depth structure activations in PIT (short arrow), in which there was no significant effect of CIP inactivation. Same conventions as in Fig 2B. *t* values in [27]: stereo_sal.img/hdr and stereo_musc.img/hdr.

Next, we analyzed the effect of reversible inactivation of CIP on the group data of the anterior IPS ROI, which consisted of the posterior and anterior subsectors of area AIP [17]. Reversible inactivation of area CIP markedly reduced the anterior IPS activation elicited by curved surfaces (T[304] = 2.6288; *p* = 0.009) but did not significantly affect the other conditions (Fig 3A). A two-way ANOVA with factors condition and inactivation (muscimol versus saline) revealed highly significant main effects of both condition (F[3,1216] = 13.91; *p* < 0.001) and inactivation (F[1,1216] = 13.98; *p* = 0.0002) but no significant interaction (F[3,1216] = 0.42; *p* = 0.7). Surprisingly, the effect of CIP inactivation was not uniform across the anterior IPS region: reversible CIP inactivation mainly reduced the curvature x disparity interaction effect in the most caudal part of the anterior IPS ROI (largely corresponding to the posterior subsector of AIP), but exerted little influence on the most rostral part of the anterior IPS ROI (compare left and right panels in Fig 3B; see also S2A Fig, left panel). For illustrative purposes, we calculated the PSC in muscimol and saline sessions on a path through the IPS. The decrease in the PSC of the interaction effect reached approximately 0.36%—which was comparable to that measured in CIP (0.4%)—in the caudal part of the anterior IPS ROI (see S2A and S3A Figs for individual monkeys). Overall, reversible inactivation of area CIP resulted in a significant (T = 2.43; *p* = 0.015) reduction in the curvature x disparity interaction effect in the ipsilateral anterior IPS (Fig 3B; for contralateral effects see also S4 Fig).

We verified that the effect of CIP inactivation on the anterior IPS was also present in each subject. S3A Fig shows the curvature x disparity interaction effect on the echo planar images (EPIs) of the three monkeys during saline (left panels) and during muscimol (right panels). In each animal, we measured a highly significant (monkey R: T[122] = 3.4252; *p* = 0.000837; monkey M: T[104] = 3.3155; *p* = 0.00126018; monkey S: T[213] = 4.4215; *p* = 1.5617^e-5^) reduction in the PSC of the interaction effect in the anterior IPS region of the inactivated (right) hemisphere (PSC values in S4 Fig). Thus, reversible inactivation of area CIP caused a robust and reproducible decrease in depth-structure-related fMRI activations in the anterior IPS, indicating a causal contribution from area CIP to the visual activations in the anterior lateral bank of the IPS that were elicited by 3D curved surfaces.

**Fig 4:**
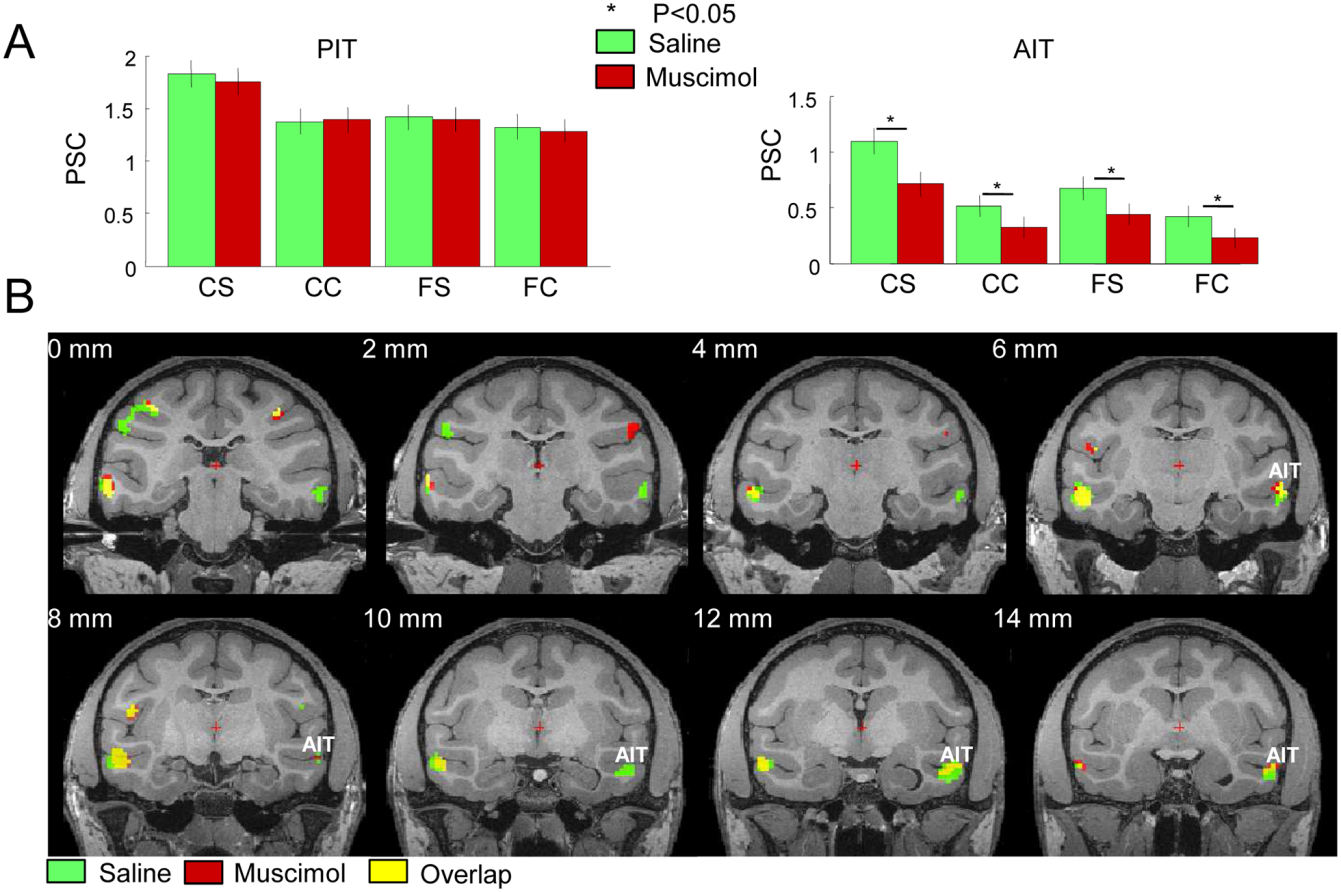
Effect of muscimol injection in CIP on the fMRI activations in the ITC. A. Group average percent signal change (compared to fixation) in the four experimental conditions in PIT (left panel) and AIT (right panel) of the inactivated (right) hemisphere. Same conventions as in Fig 2. A. * = *p* < 0.05 corrected for multiple comparisons. Raw values in [27]: AITR and PITR. B. Group average of the curvature x stereo interaction effect [(curved stereo–curved control)–(flat stereo–flat control)] in the ITC during saline and muscimol sessions, plotted on coronal sections of the M12 anatomical template (average of three monkeys; *p* < 0.05 FWE corrected). The most posterior sections (labeled “0 mm,” top left) illustrate the depth-structure-related activations in the most posterior part of the ROI of AIT (green voxels indicating a significant activation during saline but not during muscimol sessions). The ROI of PIT is shown in Fig 3B. *t* values in [27]: stereo_saline.img/hdr and stereo_musc.img/hdr.

Our second objective was to investigate the effect of CIP inactivation on the depth-structure-related activations in the ITC. In saline sessions, both PIT and AIT were significantly activated in all stereo conditions, but more so by curved surfaces (significant curvature x disparity interaction effect, group data). CIP inactivation did not affect the fMRI activations in PIT in any of the conditions (Fig 4A, left panel). However, the ROI of AIT showed a significant reduction in activation in all conditions (Fig 4A, right panel), while the most significant effect of CIP inactivation on AIT (T[304] = 4.171196; *p* = 0.0004) was observed in the curved stereo condition. The main effects of inactivation (F[1,1216] = 13.61; *p* = 0.0004) and stereo condition (F[3,1216] = 12.44; *p* < 0.0001) were highly significant (ANOVA) in the ROI of AIT, whereas the interaction between the factors inactivation and condition almost reached significance (F[3,1216] = 0.4; *p* = 0.07). As in the anterior IPS region, CIP inactivation caused a significant (T = 2.714; *p* = 0.007) decrease in the curvature x disparity interaction effect in AIT. On the group data of the curvature x disparity interaction effect illustrated in Fig 4B, it is clear that CIP inactivation reduces the depth-structure-related activation in the rostral lower bank of the STS, a part of AIT (note that Fig 4B only illustrates the depth-structure-related activations in AIT; those of PIT are shown in Fig 3B). The magnitude of the effect of CIP inactivation on AIT was surprisingly large compared to that in the IPS areas: CIP inactivation reduced the PSC of the curvature x disparity interaction effect in AIT to 52% of the level measured during saline sessions, compared to 43% for the caudal IPS region and 64% for the anterior IPS region. A two-way ANOVA of the PSC evoked by curved surfaces calculated across runs with factors area (anterior IPS versus AIT) and inactivation (muscimol versus saline), and monkey as a nested factor, showed a highly significant main effect of area (F[1,608] = 66.87; *p* < 0.0001) and inactivation (F[1,608] = 20.77; *p* < 0.0001), but no significant interaction (F[1,608] = 0.12; *p* = 0.73). As with the IPS region, we calculated the decrease in the PSC along a path through the ITC (S2A Fig, right panel) from posterior (PIT) to anterior (AIT), and found a reduction in the interaction effect induced by CIP inactivation in AIT, but not in PIT.

To demonstrate the consistency of the effect of CIP inactivation on AIT, we plotted the curvature x disparity interaction effect during saline and muscimol sessions on the EPIs of the individual monkeys (S3B Fig). In every animal, we observed a decrease in depth-structure-related fMRI activations in the ipsilateral AIT, which was significant in monkeys M (T[104] = 2.9587; *p* = 0.003825) and R (T[122] = 2.1583; *p* = 0.03) but not in monkey S (T[213] = 1.84778; *p* = 0.0660; PSC in S4 Fig). Note, however, that monkey S showed a significant effect of CIP inactivation in the more anterior part of AIT (T[213] = 2.0265; *p* = 0.043). Thus, although slightly more variable compared to the anterior IPS region, reversible CIP inactivation caused a pronounced decrease in the fMRI activations related to depth structure sensitivity in AIT, implying that CIP contributes to the depth structure sensitivity of the anterior IPS and to that of ventral stream area AIT. Finally, in order to visualize the effects of CIP inactivation on the depth structure network more directly, we subtracted the group average results for the contrast [(curved stereo–curved control)–(flat stereo–flat control)] during muscimol sessions from the same contrast during saline sessions (S3C Fig). We observed significant effects of CIP inactivation in the caudal IPS, in the anterior IPS, and in AIT, consistent with the previous analyses.

In the previous group analyses, we combined the data from equal numbers of runs from all three monkeys collected in different sessions. Although the results were consistent across subjects, we observed considerable variability between sessions. In S5 Fig, we plotted the PSC for every individual muscimol and saline session in the three main regions of interest: caudal IPS, anterior IPS, and AIT. We could reproduce the overall effect of CIP inactivation in different sessions in every animal, but remarkably, two out of ten muscimol sessions (monkey R, session 2 and monkey S, session 3; compare PSC in the current muscimol session with averaged PSC over saline sessions) did not show any effect of CIP inactivation, not even in the caudal IPS ROI. Technical factors (e.g., suboptimal injection volume, lower muscimol activity) or varying compensatory mechanisms (see discussion) may account for this intersession variability. For comparison, we also show the PSC in the same ROIs in the no-injection experiments (which were scanned months before the muscimol and saline sessions).

In saline sessions, two of our subjects (M and S) showed a significant curvature x disparity interaction effect in area F5a of the ipsilateral hemisphere. Reversible inactivation of CIP significantly reduced the depth-structure-related activations in F5a in these two monkeys (monkey M: T[104] = 3.0105; *p* = 0.003273; monkey S: T[213] = 3.2245; *p* = 0.0015). The decrease in the interaction effect in F5a of the third monkey (R) was not significant. In view of the anatomical connections between F5a and area AIP [16,17], the effect of CIP inactivation on F5a was in all likelihood a result of its effect on the anterior IPS. However, CIP inactivation did not affect every node of the depth structure network, since we observed no effect of CIP inactivation on curvature x disparity interaction in the occipital ROI comprising ventral and dorsal V2, V3, and V4 (*p* = 0.4847), nor in PIT (*p* = 0.1145) (see also S3C Fig).

We also explored whether other regions (defined on the basis of anatomical ROIs) outside the depth structure network were significantly affected by reversible inactivation of CIP. Since the main effect of disparity (contrast all stereo stimuli versus all control stimuli) produced widespread activations throughout occipital, temporal, parietal, and frontal cortex (S6 Fig), we calculated the effect of CIP inactivation on the main effect of disparity in 21 predefined anatomical ROIs in the right (inactivated) hemisphere (the effect of CIP inactivation on the main effect of disparity along a path in the IPS and in ITC is illustrated in S2B Fig). The ROI of area FST, in the fundus of the STS, was the only region outside the higher-order activations showing a significant effect of CIP inactivation on the main effect of stereo (*p* < 0.01, Bonferroni corrected for multiple comparisons). Moreover, dorsal and ventral area V4 were the only ROIs showing a significant (*p* < 0.05, Bonferroni corrected for multiple comparisons) reduction in visually-driven activations (contrast all conditions–fixation) caused by CIP inactivation. (For an overview of the effects of CIP inactivation on the contralateral hemisphere, see S4 Fig).

### The Effect of Reversible Inactivation of Area CIP on Depth Structure Categorization

**Fig 5:**
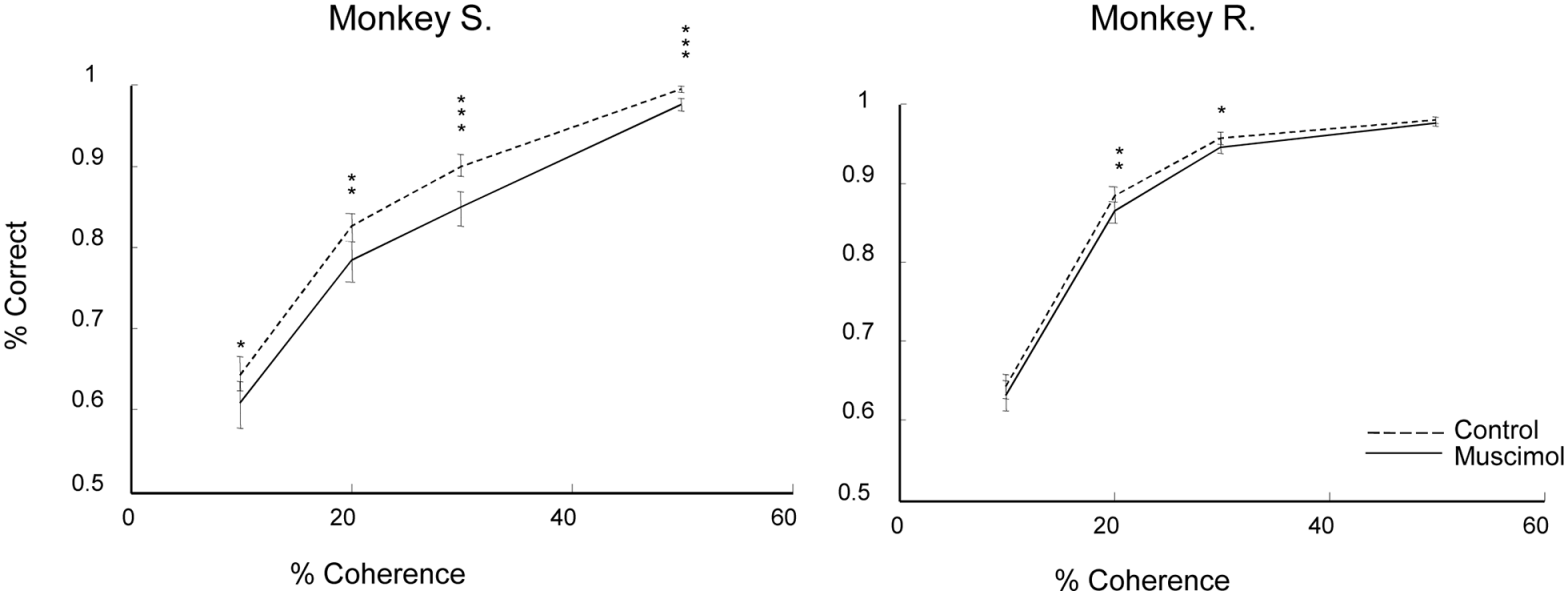
Effect of CIP inactivation on depth structure categorization. Percent correct is plotted for the four disparity coherences used (10%, 20%, 30%, and 50%) for monkey S (left panel) and monkey R (right panel). Dashed line: no inactivation; full line: CIP inactivation. The no-inactivation condition included one saline injection in monkey R. * = *p* < 0.05. ** = *p* < 0.01, ** = *p* < 0.001. Raw values in [27]; Monkey_percentcorrect.xls.

Because electrical microstimulation of the rostral lower bank of the STS, part of AIT, predictably alters the performance of monkeys in a depth structure categorization task [30], and since CIP inactivation reduced the fMRI activation in the rostral lower bank of the STS, we wondered whether CIP inactivation would also affect psychophysical performance in this task. To that end, we trained two of the monkeys (S and R) to categorize depth structure (convex versus concave) in random-dot stereograms presented at three positions in depth and at various disparity coherences (between 10% and 50% coherence). After stable psychophysical performance had been achieved, we injected muscimol in area CIP of the left hemisphere of monkey R and the right hemisphere of monkey S during depth structure categorization, in alternating sessions with saline injections (monkey R) or no injection (monkey S). At 20% disparity coherence, both monkeys showed significant decreases in percent correct during reversible inactivation of area CIP (Fig 5). The decrease in depth structure categorization performance elicited by CIP inactivation was small (monkey S: -4%; monkey R: -2%) but highly significant in both animals (monkey S: *p* = 0.001, monkey R: *p* = 0.007). In addition, monkey S was also significantly impaired at the higher disparity coherences (30%: *p* = 0.0001; 50%: *p* = 0.001). Note that the smaller effect of CIP inactivation in monkey R may have been related to the saline controls in this monkey (instead of no injection, as in monkey S). We also plotted the percent concave responses as a function of percent coherence to investigate the presence of a general bias in the animals' responses (S7A Fig). The behavioral data were fitted using logistic regression. In both monkeys, the slope of the fitted psychometric function was significantly less steep in muscimol sessions (difference in slope for monkey S = 0.156, *p* = 0.005, and for monkey R = 0.108, *p* = 0.004) compared to control sessions, but no response bias was found.

Consistent with a previous study [24], the mean horizontal eye position traces, which indicate commitment of the monkey to a specific choice, began to differ for convex and concave choices at 220 ms (monkey S) and 300 ms (monkey R) after stimulus onset in the absence of muscimol (saline or no injection, ROC analysis, S7B Fig). The difference in eye positions appeared 20 ms (monkey S) and 10 ms (monkey R) later when CIP was inactivated (S7C Fig). Similarly, CIP inactivation slightly delayed the response times (i.e., the time between stimulus offset and saccade onset) by 18 ms in monkey S (two-way nested ANOVA with factors session and saline/muscimol, F = 7.99; *p* = 0.02) and by 10 ms (ANOVA, F = 436.15; *p* < 0.001) in monkey R. Thus, a single unilateral injection of muscimol in area CIP resulted in a significant degradation of performance in a depth structure categorization task.

### CIP Microstimulation during fMRI Activates a Distinct Network of Cortical Areas

**Fig 6:**
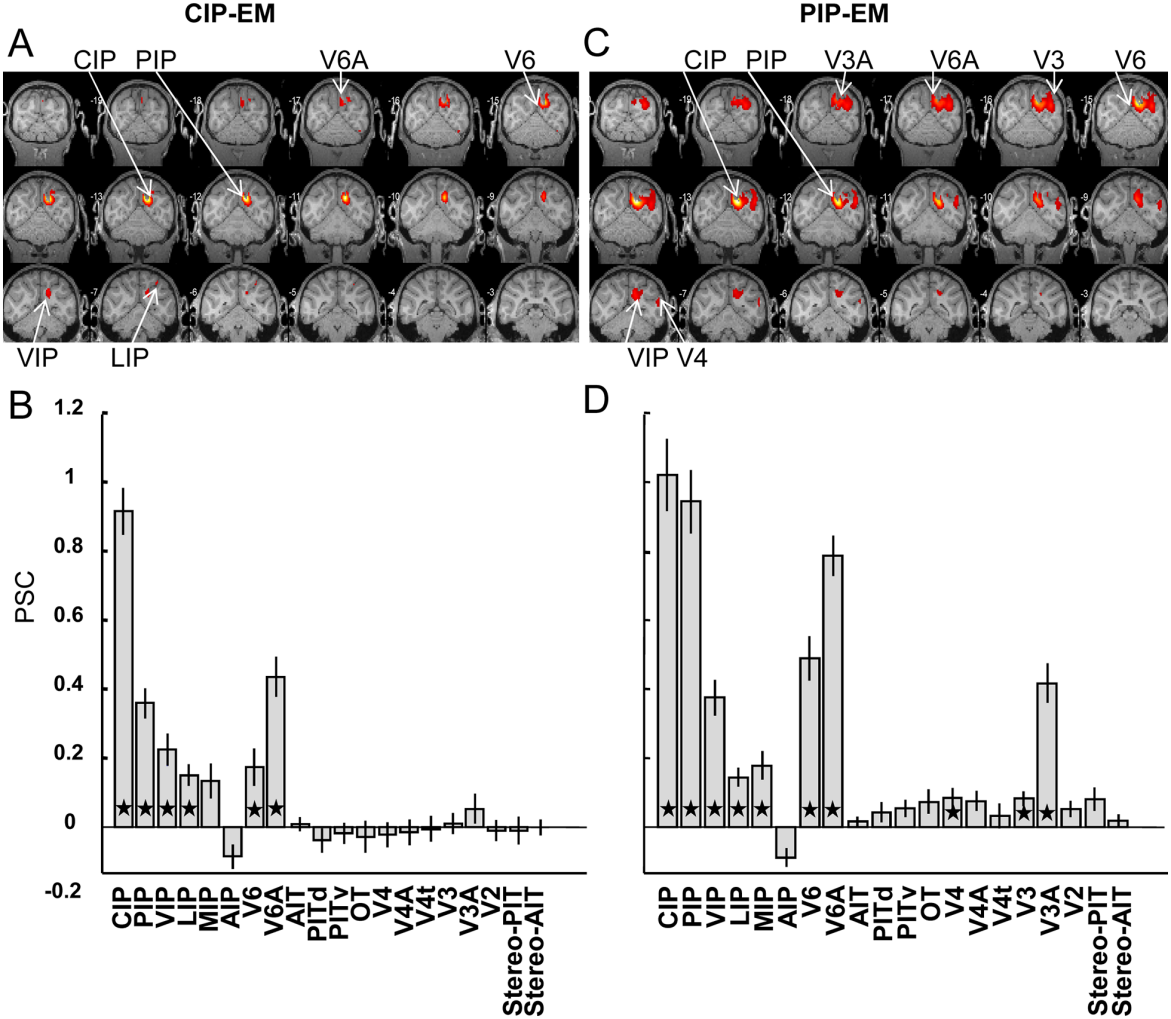
Effective connectivity of CIP and PIP. A. EM-induced fMRI activations during stimulation of CIP, plotted on coronal sections of the M12 anatomical template (average of two monkeys at *p* < 0.05 FWE corrected). B. Percent signal change induced by CIP-EM (compared to no stimulation) in 18 anatomical ROIs and two functionally-defined ROIs (stereo-PIT and stereo-AIT). Error bars indicate standard error across runs. Black asterisks indicate significant increase in PSC compared to no stimulation (*t* test, *p* < 0.05, corrected for multiple comparisons). C. EM-induced fMRI activations during stimulation of PIP plotted on coronal sections of the M12 anatomical template (average of three monkeys). D. Percent signal change induced by PIP-EM in the same 18 anatomical and two functionally-defined ROIs (stereo-PIT and stereo-AIT). See [27]; *t* values in CIP-EM.img/hdr and PIP-EM.img/hdr; PSC in PSC@CIP_EM.xls and PSC@PIP_EM.xls.

To determine whether CIP is effectively connected to AIT, we electrically stimulated CIP during fMRI in two monkeys (four sessions/30 runs in monkey M, who was also used in the inactivation experiments, and four sessions/36 runs in monkey D). In monkey M, we stimulated at the center of the stereo-activation in the lateral bank of the caudal IPS, whereas in monkey D, we stimulated in a site in the lateral bank of the caudal IPS where we recorded selective responses to planar disparity-defined surfaces (S8A Fig). In both animals, CIP-EM elicited strong activations (Fig 6A, *p* < 0.05 FWE corrected) in the IPS areas PIP (in the medial bank of the caudal IPS), VIP, and in the most posterior (dorsal) part of LIP, but also in the medial parietal areas V6 and V6A (Fig 6B, one-tailed *t* tests on PSC: *p* <0.05, Bonferroni corrected for multiple comparisons). Results for the two monkeys were quite similar (see S8C Fig for individual results). However, no significant fMRI activations were observed in any of the temporal ROIs, nor in parietal area AIP (Fig 6A). We verified the absence of any increase in PSC in the functionally-defined ROIs of PIT and AIT (Fig 6B, rightmost bars).

Because CIP-EM strongly activated area PIP in the medial bank of the caudal IPS, we also stimulated area PIP in three monkeys (R and M from the CIP inactivation experiment: five sessions/40 runs and three sessions/34 runs respectively, and D from the CIP-EM experiment: three sessions/31 runs). The location of the stimulation site was determined by the depth-structure activations in the fMRI experiment (in monkeys M and R), and by single-cell selectivity for disparity-defined planar surfaces (in monkey D and R, example neuron in S8B Fig). In parietal cortex, the pattern of activations elicited by PIP-EM was highly similar to that evoked by CIP-EM (Fig 6C and 6D): the anatomical ROIs of areas CIP, VIP, V6, and V6A were significantly activated by PIP-EM (one-tailed *t* tests on PSC: *p* < 0.05, Bonferroni corrected for multiple comparisons). However, in contrast to CIP-EM, we also observed strong activations in area V3A and (weaker) activations in areas V3 and V4. Nonetheless, PIP-EM did not activate the more anteriorly located AIT, nor the anatomical ROI of AIP. We verified the lack of increased PSC in the functionally-defined ROIs of PIT and AIT (Fig 6D, rightmost bars). Thus, the reduction in depth-structure-related activations in AIT and the anterior IPS consequent to CIP inactivation were not paralleled by EM-elicited activations in these regions.

## Discussion

To elucidate how the dorsal and ventral stream interact during 3D object vision, we capitalized on the recruitment of posterior parietal and inferotemporal regions during fMRI when viewing 3D stimuli. We report here that area CIP contributes causally to the 3D object-related fMRI activations in the anterior IPS and, surprisingly, also to those in AIT, one of the end-stages of the ventral visual stream. Moreover, CIP inactivation significantly affected performance in a depth-structure categorization task. In contrast, electrical microstimulation of CIP during fMRI activated neither AIT nor the anterior IPS. To our knowledge, these results represent the first evidence that 3D object information in the dorsal stream affects visual processing in the ventral visual stream.

To our knowledge, this is the first study in which fMRI, reversible inactivation, electrical microstimulation, and behavioral measurements were combined to investigate the impact of a cortical area (CIP) on other nodes of the network in which it is embedded. EM-fMRI can reveal the effective connectivity of patches of neurons with similar properties within a cortical area [17,31,32], but cannot easily determine which information is being transferred between the different nodes of the network. Here, we measured the effect of CIP inactivation on the fMRI activations elicited by different visual stimuli (curved and flat surfaces) in posterior parietal and inferotemporal cortex, so that we could infer which aspect of visual processing was affected in these remote areas. Thus, inactivation-fMRI in combination with different visual stimulus conditions can clarify in a much more detailed way how cortical networks operate, above and beyond effective connectivity.

In contrast to previous studies [20,22], we observed robust activations related to depth structure sensitivity in the caudal IPS, in area PIT (partially corresponding to TEO [26]) and in AIT. The most anterior fMRI activation in AIT was located in the rostral lower bank and shoulder of the STS, in line with previous single-cell studies [21,28]. Undoubtedly, technological improvements (3T scanner, 8-channel coil, and a 4-fold higher spatial resolution) have contributed to this result. Moreover, in contrast to the previous studies, all monkeys in the present study were first trained to discriminate depth structure in curved surfaces [24]. Hence, the combination of higher signal-to-noise, higher spatial resolution, and stringent behavioral control most likely explains the discrepancy between our fMRI results and previous studies. It should be noted that our fMRI results identified the activation in PIT as a very likely second—and possibly even more important—input stage for AIT, consistent with previous anatomical studies [33,34]. Thus, the depth structure-sensitive part of AIT that subserves depth structure categorization [30] may receive multiple inputs from both dorsal and ventral visual streams.

Our results are also relevant for the functional organization of the human visual cortex and human–monkey homologies. Several studies [35–37] have identified a region in the caudal IPS in humans that is activated strongly by stimuli that are known to activate CIP neurons in monkeys (planar surfaces in depth), and [4] showed stronger fMRI activations elicited by near/far disparity compared to zero disparity in the monkey CIP and in the human caudal IPS. Note that the fMRI activations in the human caudal IPS are frequently located more medially in the caudal IPS, whereas the monkey CIP is located in the caudal lateral bank of the IPS. However, in our study, both the lateral (CIP) and the medial (PIP) bank of the IPS were more activated by curved surfaces than by flat surfaces. Similarly, numerous studies have investigated the putative human homologue of area AIP using fMRI, e.g., [4,18,38–41]; for a discussion on human and monkey AIP, see [10]. In addition, it has been proposed [10,36,42] that the visual object responses in the anterior IPS depend on 3D information represented in CIP, and, to our knowledge, our study provides the first causal evidence that this is indeed true. Finally, the strong depth-structure-related activation in PIT (partially corresponding to TEO) on the lip of the STS is a new finding (previous research showed activations for near–far disparities in a different part of PIT, in the fundus of the STS [3]) that may be highly relevant to the organization of the human occipitotemporal cortex. Using the same stimuli as in the current study, [40] observed a single ventral stream fMRI activation in the human lateral occipital complex (LOC), which was interpreted as potentially homologous to the monkey AIT, because [22] also observed a single fMRI activation in AIT of the monkey. However, because of the susceptibility artefact (loss of fMRI signal) in the human temporal lobe, it was difficult to determine whether more anterior regions in occipitotemporal cortex were also sensitive to depth structure. In the current study, we measured a much more extensive network in the monkey ITC, with significant depth-structure-related fMRI activations in PIT and in AIT. Hence, the LOC activation in humans may be homologous to the monkey PIT.

We targeted CIP because of the significant depth-structure-related activation in the caudal IPS in our first fMRI experiment, and because [4] has also reported strong fMRI activations evoked by near–far disparities in CIP. Moreover, electrical microstimulation of the most posterior subsector of AIP (pAIP) during fMRI activates both CIP and PIP [17]. Earlier single-cell studies had demonstrated that CIP neurons respond selectively to the 3D orientation of planar surfaces defined by binocular disparity [43,44] and other depth cues [45,46] and, to some extent, even to curved surfaces [47]. Hence, CIP may be an important—and possibly even the first—processing stage in the dorsal stream for the computation of changes in disparity along a surface, as opposed to absolute or relative disparity, which are represented—at least at the single-cell level (see [48])—at earlier stages in the visual hierarchy such as areas V2 [49], V3, and V3A [50].

Several observations argue against an attentional interpretation of our inactivation results. First, if CIP inactivation were to indirectly (through LIP) reduce attentional gain in different parts of cortex, this gain change would not be specific to the 3D-shape interaction contrast. Indeed, one would expect lowered activity for all ipsilateral visual activations. However, although we observed widespread activations to disparity (contrast all stereo versus all control stimuli) throughout visual cortex, CIP inactivation resulted in limited decreases in disparity-induced activity: the ROI of area FST was the only region (out of 21 predefined anatomical ROIs in the right/inactivated hemisphere) outside the areas with higher-order activations showing a significant reduction in disparity activation following CIP inactivation. Furthermore, despite widespread visual activations to stimuli in the entire stimulus set (contrast all conditions-fixation), CIP inactivation only caused reductions in visually-driven activity in area V4. Thus, CIP inactivation did not result in global (ipsilateral) gain reductions in visual cortex. Instead, CIP inactivation caused highly specific, 3D-structure-related, activity reductions. Given that 3D-structure-related areas contribute to 3D-structure discrimination performance (e.g., [30]), it seems likely that these targeted 3D-structure-related activity decreases had an adverse effect on the monkeys' performance during 3D-structure categorization, as supported by our data. Finally, even though we cannot entirely exclude an attentional interpretation, our main conclusion remains that reversible inactivation of a visual area in the dorsal stream (CIP) affects visual activations in the ventral stream.

The effect of reversible inactivation of CIP on remote areas could be either direct (through direct cortico-cortical connections) or indirect (through other areas). The clearest example of an indirect effect was the decrease in fMRI activations in F5a after CIP inactivation, which must have been a result of its effect on AIP, with which F5a is connected and shares many properties [17,51,52]. Previous studies have shown that EM-fMRI furnishes an almost exact replica of the anatomical connectivity of an area [17,31,32]. Here, CIP-EM did not activate AIT nor any other ventral stream area, indicating that the effect of CIP inactivation on AIT was most likely indirect. This assertion is supported by anatomical connectivity studies, which have revealed that CIP connects primarily with other areas in the IPS such as LIP and (to a lesser extent) AIP [53], and only very weakly with AIT [54]. Since depth-structure-selective patches in pAIP are effectively connected with AIT [17], the reduction in fMRI activations in AIT caused by CIP inactivation was most likely established by means of its effect on AIP. These results clearly illustrate that EM-fMRI complements inactivation-fMRI when interpreting direct and indirect effects of reversible inactivation.

**Fig 7:**
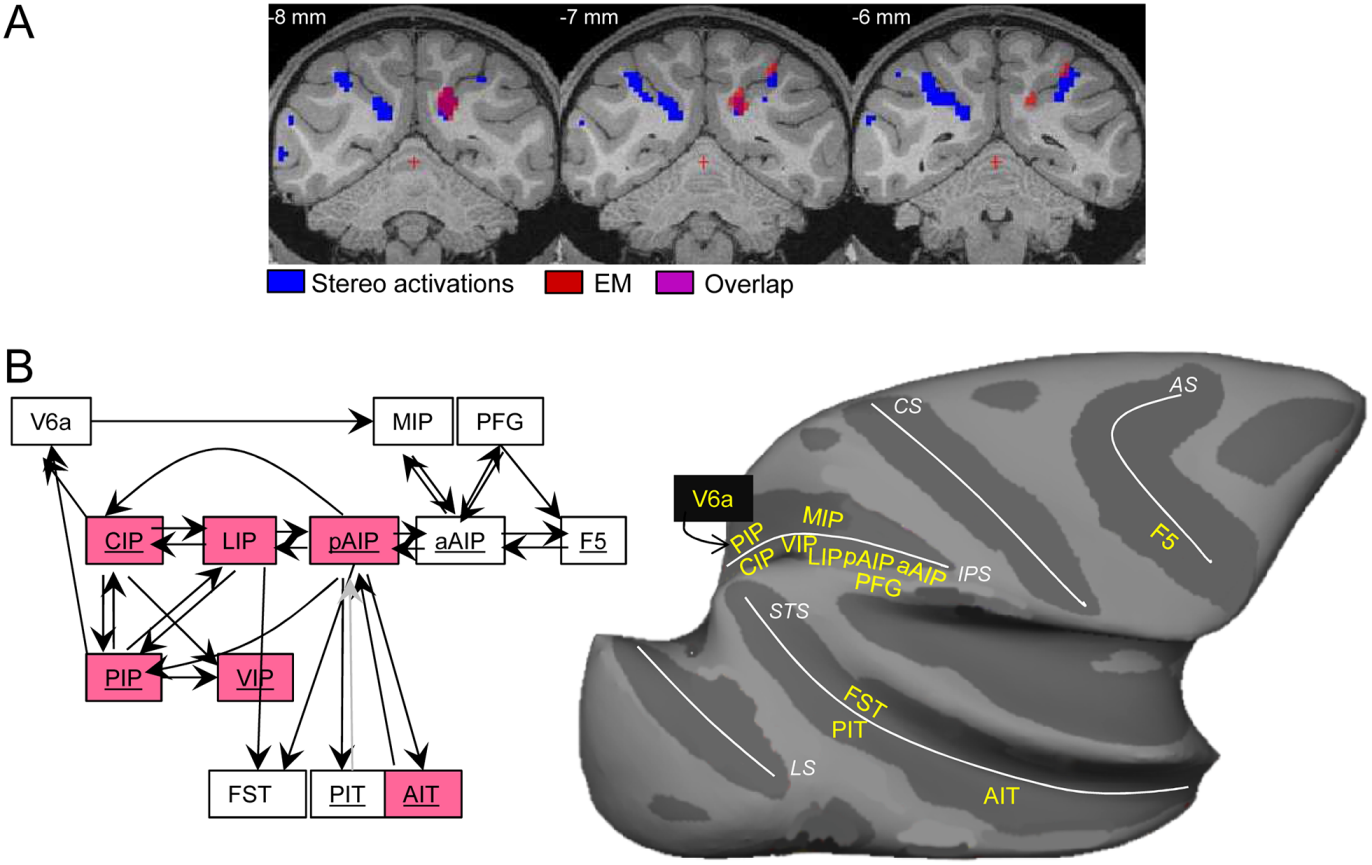
Overlap of depth structure activations and CIP-EM activations in the caudal IPS. A. Depth structure activations (of monkey M, blue) and CIP-EM induced activations (red) are plotted on coronal slices of the M12 template. Overlap is indicated by the dark red color. See [27]; *t* values in stereo.img/hdr and CIP-EM.img/hdr. B. Schematic diagram of known functional interactions between dorsomedial, dorsolateral, and ventral visual streams, based on the results of this and a previous study. Boxes in red indicate areas or regions significantly affected by CIP inactivation, and underlined area names indicate significant depth structure activations. Dark arrows indicate effective connectivity based on anatomical tracer and/or EM-fMRI studies. The gray arrow from PIT to pAIP is presumed connectivity based on the bidirectionality of most cortico-cortical connections. C. Inflated macaque brain displaying the main cortical areas implicated in stereo processing, and their connected areas. IPS: intraparietal sulcus; CS: central sulcus; AS: arcuate sulcus; LS: lunate sulcus; STS: superior temporal sulcus.

Although caution is warranted (since the absence of an EM-induced fMRI activation does not rule out the presence of a direct anatomical connection), the absence of anterior IPS activations during CIP-EM suggests that the CIP-inactivation effects on the anterior IPS were also indirect. To determine how CIP may have affected pAIP, we mapped the CIP-EM-induced activations onto the depth-structure-related activations of monkey M, which was the only animal involved in both the CIP-EM experiments and the stereo fMRI experiments. The overlap between these two types of activations in both dorsal LIP and VIP (Fig 7A) suggests that the CIP-inactivation effect on the anterior IPS was mediated through (dorsal) LIP and/or VIP. Moreover, the depth-structure activations at these anterior–posterior levels in the IPS were significantly reduced after CIP inactivation (S9 Fig). These results are in agreement with a previous anatomical tracer study [55] showing that CIP is connected to area AIP through a series of projections along the lateral bank of the IPS.

We did not activate pAIP when stimulating CIP, although weak direct CIP–AIP connections have been described [16,55]. Hence, EM-fMRI may primarily reveal the strongest anatomical connections of a given cortical area. Alternatively, our EM-fMRI experiments were more specific than anatomical tracer studies, since we selectively targeted patches within CIP selective for higher-order disparity. Since microstimulation in depth-structure-selective patches in pAIP activated both CIP and PIP [17], the possibility exists that, even at this level of the visual hierarchy, cortico-cortical connections may be unidirectional, in line with recent anatomical studies [54].

The EM-fMRI experiments clearly demonstrated that CIP and PIP are strongly interconnected, but the pattern of PIP connectivity outside parietal cortex differed markedly from that of CIP. Only PIP-EM activated the early visual areas V2, V3, and V3A. Moreover, the CIP and PIP EM-fMRI experiments have revealed, to our knowledge, for the first time how the depth structure network, which is confined to the dorsolateral and ventral visual stream, is connected to areas belonging to the dorsomedial stream [56–58]: both CIP and PIP-EM elicited strong activations in the anatomical ROIs of areas V6 and V6A. Recent studies suggest that V6A neurons respond selectively to different grip types and even to different objects [59,60], similar to neurons in AIP [61]. Hence, our data also reveal the pivotal role played by CIP, where the dorsolateral and dorsomedial pathways intersect when processing objects for grasping.

We demonstrated the causal effect of CIP inactivation upon the anterior IPS region and AIT by means of fMRI, which represents an indirect measure of neural activity based on changes in blood oxygenation [62,63]. Hence, our results cannot determine what the effect of CIP inactivation may be on the neural responses in these higher-order areas. In theory, CIP inactivation may merely evoke subthreshold modulations in its target areas (possibly measurable at the level of the local field potential) without affecting the neuronal firing rate or selectivity. However, the perceptual effect of CIP inactivation on depth structure categorization must presumably have altered the firing rates of neurons at some distance from the injection site. Since electrical microstimulation of AIT neurons has a profound and predictable effect on depth structure categorization [30], the most likely route through which CIP inactivation influenced depth structure categorization is through AIT. It is important to emphasize that a number of single-cell studies have already characterized the depth structure selectivity of individual neurons in almost all nodes of this network in great detail: in AIT [21,24,28,30,64], AIP [23,25,65], F5a [29,52], and CIP [47]. Despite the inherent assumptions associated with fMRI [25], these single-cell studies provide strong support for the notion that the curvature x disparity interaction contrast we used is effectively an fMRI indicator of higher-order disparity sensitivity at the neuronal level.

The small but highly significant effect of CIP inactivation on accuracy in a depth-structure categorization task was similar in magnitude to that reported in a recent inactivation study in the ITC [66]. Several factors may explain why we did not observe larger effects in this task. We used a fixed-duration paradigm with a relatively long stimulus duration (800 ms), whereas previous studies [30,67] have reported reaction times of less than 300 ms in a reaction-time version of the same task. Hence, our long stimulus duration may have allowed time for additional processing of the stimulus. In addition, we observed residual depth-structure-related activations in the caudal IPS after muscimol injection, indicating that we may have only partially inactivated CIP. Note also that a previous CIP inactivation study observed a significant deficit in the discrimination of surface orientation in only three out of six injections [45]. More importantly, CIP inactivation did not significantly alter the activations in PIT, nor in earlier visual areas such as V3 and V4. Thus, our muscimol injection in CIP may have affected only a small portion of the extensive depth structure network, such that other areas (possibly in the contralateral hemisphere as well) remained functional. A previous human fMRI study [68] showed that parietal activations correlate negatively with disparity coherence and psychophysical performance during 3D shape judgments. Hence, especially at lower disparity coherences, compensatory mechanisms may emerge that can obscure the effect of reversible inactivation.

To our knowledge, our study is the first systematic attempt to dissect the neural circuitry subserving 3D object vision by means of an integrated approach combining fMRI, reversible inactivation, electrical microstimulation, and single-cell recordings. Fig 7B and 7C summarizes the main findings of this and a previous EM-fMRI study [17]. Despite the extreme interconnectedness of the cortical circuitry, it becomes possible to discern how 3D object information may be transmitted throughout the dorsolateral stream, from the relatively early stages in areas CIP and PIP, through LIP, pAIP, and aAIP to the motor system, and where these areas interact with the dorsomedial and the ventral stream. Thus, progress in our understanding of the neural basis of 3D object vision provides a glimpse into the intricate organization of, and functional interactions within, large-scale cortical networks throughout visual cortex, including their target areas in the motor system.

## Methods

### Subjects, Surgery, and Stimuli

In total, five male rhesus monkeys (monkey K: 4 kg, 4 y old; monkey M, 6 kg, 5 y old; Monkey R: 5 kg, 6 y old; Monkey S: 4 kg, 5 y old; Monkey D: 7 kg, 11 y old) participated in the present experiments.

Animal housing and handling were in accordance with the recommendations of the Weatherall report, allowing locomotor behavior, social interactions, and foraging. All animals were pair- or group-housed (two to four animals per group; cage size at least 16–32 m^3^) with cage enrichment (toys, swings, foraging devices) at the primate facility of the KU Leuven Medical School. The natural light/dark cycle was followed, and all experiments were performed during daytime (between 8 AM and 8 PM). Animals were fed daily with standard primate chow supplemented with bread, nuts, raisins, prunes, and fruits. The animals received their daily water supply during training and experiments, or ad libitum in the cages in between training or experimental periods.

Monkeys K, M, R, and S were previously not used in other studies; monkey D was enrolled in other electrophysiological studies prior to this one.

Animal care and experimental procedures complied with the national and European guidelines (Directive 2010/63/EU) and were approved by the Ethical Committee of the KU Leuven (project number P063/2010). No animals were sacrificed for this study, and except for monkey M, all animals are currently enrolled in other experiments. The implant of monkey M has been removed to prepare the animal for retirement.

Each monkey was implanted with an MRI-compatible head fixation post on the skull using ceramic screws and dental acrylic using isoflurane anesthesia and under sterile conditions. Six w after surgery, the monkeys were trained to maintain fixation upon a spot inside a 1.5°, electronically-defined window while 3D stimuli were presented foveally. After adequate performance in passive fixation had been reached, all animals were trained in a depth structure categorization task [16].

The stimulus set consisted of curved and flat random-dot stereograms in which depth was defined by horizontal disparity (dot size 0.08°, dot density 50%, vertical size 5.5°), presented on a grey background. All stimuli were generated using Matlab (R2010a, MathWorks) and were gamma-corrected. Similar to [20] and [22], we used a 2-by-2 design (S1A Fig) with factors curvature (curved versus flat) and disparity (stereo versus control). In the inactivation-psychophysics experiments and during training, dichoptic presentation of the stimuli was achieved by means of a double pair of ferroelectric liquid crystal shutters (DisplayTech) operating at 60 Hz each. The shutters opened and closed in synchrony with the vertical retrace of the display monitor (VRG, P46 phosphor, vertical refresh rate is 120Hz). There was no measurable cross-talk between the two eyes [23]. The position of the right eye was recorded by means of an infrared-based camera system sampling at 500 Hz (EyeLink 1000, SR Research). Simultaneously with the stimulus presentation, a bright square at the right bottom of the display (invisible to the monkey) was presented, detected, and registered as photocell pulses by a photodiode attached to the display. The photocell pulses and the recorded eye movements were digitized and processed at 20 kHz on a single digital processor (DSP, C6000 series, Texas Instruments). In the fMRI experiments, red/green versions of the stimuli were used, and red/green stereoglasses were placed before the eyes to provide dichoptic presentation of the stimuli.

The stereo-curved condition consisted of three types of smoothly curved depth profiles (1, ½, or ¼ vertical sinusoidal cycle) together with their antiphase counterparts obtained by interchanging the monocular images of the two eyes (disparity amplitude within the surface: 0.5°). Four different circumference shapes (example in S1 Fig) were combined with each of these six depth profiles, and each combination could appear at two different positions in depth (mean disparity +/– 0.5°), creating a set of 48 curved surfaces. In the stereo-flat condition, flat surfaces (using the same four circumference shapes) were presented at 12 different positions in depth, such that the disparity content was identical to that in the stereo curved condition. Finally, in the control conditions, we presented one of the monocular images (either belonging to one of the stereo curved stimuli of to one of the stereo-flat stimuli) to both eyes simultaneously (curved-control and flat-control). These control conditions consisted of the same binocular input as the stereo conditions, since they contained exactly the same monocular images as the corresponding stereo condition.

In the inactivation–psychophysics experiments, the stimuli were double-curved (along the horizontal and vertical axes) convex and concave surfaces with a circular circumference shape, and were presented with different disparity coherences (10%–50%), as in [24].

### Training in Depth-Structure Categorization

Monkeys had to indicate the perceived depth structure of a random-dot stereogram (presented at the fixation point for a fixed duration of 800 ms) by means of an eye movement (to the left for concave or to the right for convex) in order to obtain a liquid reward. The depth structure of the random-dot stereogram was solely defined by horizontal disparity as a two-dimensional radially-based Gaussian surface that could be either convex or concave. In order to encourage discrimination of the 3D-profile rather than absolute disparity, the 3D stimuli were presented at three different positions in depth (near, at the fixation plane, and far). Since the disparity gradient was present only along the surface of the stimulus and not at the boundary [65], monocular cues were absent. Task difficulty was manipulated by varying the percentage of dots defining the surface, i.e., the disparity coherence. A random disparity (drawn from a uniform distribution [-0.5 degrees, 0.5 degrees]) was applied to dots that were not enclosed in the surface. All stimuli contained the same number of dots, regardless of the disparity coherence or position in depth of the 3D-shape. The contours of all 3D stimuli were circular. Monkeys K and M were trained until they reached a performance of 80% correct for a 100% disparity coherence stimulus (typically after 3 to 4 w of training), whereas monkeys R and S were trained with disparity coherences between 100% and 10% until they performed above chance level at the 10% disparity coherence stimuli.

### Scanning Procedures

Four monkeys (M, K, S, and R.) were trained to sit in a sphinx position in a plastic MRI-compatible monkey chair positioned in the horizontal bore of the magnet. The stimuli were rear-projected from a Barco 6300 LCD projector onto a translucent screen in front of the monkey at a 57 cm distance. Red/green stereoglasses were placed before the eyes to provide dichoptic presentations of the stimuli. A pupil corneal reflection tracking system (Iscan) was used to monitor the position of the eye at 120 Hz during scanning. Maintenance of fixation within the 1.5° electronically-defined window around the fixation point was required in order to be rewarded. The inter-reward-interval was systematically decreased (from 2,000 to 900 ms) when the monkey maintained fixation within the window, to encourage long, uninterrupted sequences of fixation. Prior to the scanning sessions, a contrast agent (monocrystalline iron oxide nanoparticle [MION]; Feraheme, AMAG pharmaceuticals) was injected to enhance the signal-to-noise ratio [69,70] and spatial selectivity of the MR signal [71]. BOLD signals depend on a combination of cerebral blood volume (CBV), blood flow, and oxygen extraction, whereas MION measurements depend solely on CBV. Since an increase in brain activation produces a decrease in MR signal in MION CBV maps, the polarity of all signal-change values was inverted to account for differences between MION CBV and BOLD activation maps. A radial transmit-only surface coil and custom-built eight-channel phased-array receive coils were positioned closely around the monkey’s head. Functional images were acquired with a 3.0 Tesla full body scanner (TIM Trio, Siemens), using a gradient-echo single-shot T2*-weighted echo-planar imaging sequence (40 horizontal slices, TR = 2 s, TE = 17 ms, 1.25 mm isotropic).

Data were acquired using a block design whereby each block (or condition, each 24 s long) consisted of 12 functional volumes embedded in a time series of 222 volumes (444 s). Stimulus frequency was 1 Hz, and each condition (block) was repeated twice within each time series. The presentation order of the conditions was pseudo-randomized. Voxels showing a significant interaction between the factors curvature and disparity, i.e., where (curved-stereo versus curved-control) was greater than (flat-stereo versus flat-control), were deemed to be sensitive to the depth structure of surfaces [20].

### Reversible Inactivation Procedures

After the first fMRI-experiments, a recording chamber was implanted vertically above the right CIP, under isoflurane anesthesia and aseptic conditions. The position of the recording chamber was centered on the local maximum of the fMRI activation in the posterior part of the IPS at Horsley-Clarke coordinates between 4 and 8P, and between 8 and 12L for the three monkeys. To target the activated voxels in area CIP in the lateral bank of the posterior IPS showing a significant interaction between curvature and disparity, we first obtained an anatomical MRI (resolution 0.6 mm isotropic) using glass capillaries filled with a 2% copper sulfate solution inserted into several positions of a standard recording grid (Crist Instruments, spacing 1 mm) placed inside the recording chamber. The fMRI activation maps were then warped onto this MRI template. After determining the anterior–posterior and medial–lateral position in the grid, we calculated the estimated depth of the injection sites and verified this by means of an anatomical MRI that was obtained after an injection of the contrast-agent Dotarem (4 μl of a 2% solution) using a 10 μl Hamilton syringe, as shown in S1B Fig. To minimize damage to the cortex of the lateral bank of the IPS, we inserted the tip of the needle until reaching the transition between white and gray matter, so that the solution could spread to the neighboring cortex. At least one week later, we started the inactivation-fMRI experiments. In each muscimol session, 4 μl of the GABA-A agonist muscimol (Sigma, 10 mg/ml) was injected with a 10 μl Hamilton syringe connected to a 33-gauge stainless needle. The injection of muscimol always took place immediately preceding (less than 30 min) the fMRI measurements and outside the scanner. The syringes were inserted into a stainless-steel guiding tube that was placed inside the grid. The volume of the micro-infusions at each site was slowly delivered in small steps of 1 μl every minute to avoid pressure damage. Each muscimol scanning session alternated with a control saline scanning session, in which an equal amount of saline was injected at the same site prior to the scanning sessions. Saline sessions were separated from muscimol sessions by at least 24 h. After the injection of either muscimol or saline, we ran the standard fMRI experiment as described earlier. We collected a total of 219 muscimol runs and 227 saline runs, in 10 sessions each. The numbers of runs were equalized for saline and muscimol sessions within each monkey (monkey M, 51 runs; monkey S, 107 runs; monkey R, 61 runs), and over monkeys for the group analysis by excluding runs randomly, yielding 51 runs per monkey for saline and 51 runs per monkey for muscimol sessions.

We followed the same procedures when testing the effect of reversible inactivation of CIP on depth structure categorization. However, in these behavioral experiments, four disparity coherences were used (10%, 20%, 30%, and 50% coherence, convex and concave surfaces), and muscimol sessions were interleaved with two control sessions without (monkey S) or with (monkey R) saline injections.

### Electrical Microstimulation

All procedures have been described elsewhere [17]. Briefly, in every EM-fMRI session (monkeys M, D, and R), a Platinum/Iridium electrode (impedance 50–200 kΩ in situ, FHC, Bowdoinham, ME) was inserted in the grid through glass capillaries serving as guide tubes (FHC, Bowdoinham, ME). A platinum wire served as ground. To verify the stimulation positions, structural MR images (0.6 mm resolution) were acquired in every scan session (prior to the start of the fMRI experiment) while the electrode was located at the exact stimulation site inside a standard recording grid (Crist Instruments, Hagerstown, MD; S8 Fig). During EM-fMRI sessions, all animals were sedated using a 0.25/0.5 cc mixture of ketamine (Nimatek) and medetomidine (Domitor) (administered every 45 min). The animals were video-monitored during sedation, and body temperature was maintained using a heating pad. Note that we recently demonstrated highly comparable EM-fMRI effects in sedated and awake states in area AIP [17]. Using sedated animals, we can exclude any possible influence of attentional state. In every EM-fMRI session, the EM signal was produced using an eight-channel digital stimulator (DS8000, World Precision Instruments) in combination with a current isolator (DLS100, World Precision Instruments). We switched between stimulation and no-stimulation blocks (each lasting 40 s), for a total duration of 480 s. During stimulation blocks, a single EM train was applied (on average) every 3 s. Stimulation trains lasted 250 ms and were composed of biphasic square-wave pulses (repetition rate 200 Hz; amplitude 1 mA). Each EM pulse consisted of 190 μs of positive and 190 μs of negative voltage, with 0.1 ms between the two phases (total duration: 0.48 ms). The timing of the EM pulses during the fMRI experiment was computer-controlled.

### Data Analysis

Correction for body-motion artifacts was performed with an off-line SENSE (sensitivity encoding) reconstruction of the images [72]. Data were analyzed using statistical parametric mapping (SPM5) and BrainMatch software, using a fixed-effects GLM. Spatial preprocessing consisted of realignment and rigid coregistration with a template anatomy (M12) [73]. To compensate for echo-planar distortions in the images as well as inter-individual anatomical differences, the functional images were warped to the template anatomy (M12) using non-rigid matching BrainMatch software. The algorithm computes a dense deformation field by the composition of small displacements, minimizing a local correlation criterion. Regularization of the deformation field is obtained by low-pass filtering. The functional volumes were then resliced to 1 mm^3^ isotropic and smoothed with an isotropic Gaussian kernel (full width at half maximum: 1.5 mm). To avoid higher-order distortion, a nonrigid slice-by-slice distortion correction was applied to fit a fixed-effect general linear model (GLM). We used a fixed-effects analysis (as is common in monkey fMRI experiments [74,75]) because our sample size was too small for effective power in a random effects analysis. SPM5 was used to perform a voxel-based analysis as previously described [31]. To improve the GLM, six motion-realignment parameters and two eye-movement parameters were added as covariates of no interest. Eye traces were thresholded within the 1.5° (horizontally) x 2° (vertically) window, convolved with the MION response function and subsampled to the TR of 2 s. The functional volumes were resliced to 1 mm^3^ isotropic and smoothed with an isotropic Gaussian kernel (FWHM: 1.5 mm).

For illustrative purposes (Fig 1A), the SPM5 activation maps of the group analysis were plotted on flattened or coronal representations of the M12 anatomical template, using Caret software (version 5.64; http://brainvis.wustl.edu/wiki/index.php/Caret:About). Individual activation maps were plotted on the coronal slices of an averaged EPI image of each monkey, to minimize warping errors. In a manner analogous to previous studies [69,70,76,77], depth structure sensitivity was defined by a significant interaction (*p* < 0.05 FWE rate corrected, FWE for multiple comparisons on the entire brain) between the factors curvature (curved or flat) and disparity (present or absent) in the block design. Group data were analyzed as fixed effects with an equal number of volumes per monkey, supplemented with single-subject analysis.

To illustrate depth-structure sensitivity along the IPS and along the ITC in individual animals, we defined two paths along the IPS and along ITC, and then plotted the percent signal change of the curvature x disparity interaction effect in each point along the path [20,22]. The path was determined manually on the group data to include, insofar as possible, all activations related to depth structure. Therefore, one point was marked in coronal sections every millimeter, in the center of the activations, along the IPS or STS.

For the analysis of the effects of CIP inactivation on the curvature x disparity interaction in each individual animal, we determined the borders of the ROIs based on the activated clusters in the interaction-contrast T-map of the control saline scanning sessions for each animal (on the average EPI), at uncorrected level for monkey M and R (*p* < 0.001), and at corrected level for monkey S (FWE, *p* < 0.05). As in the group analysis, we identified two regions in the IPS (anterior IPS and CIP/PIP) and two regions in the superior temporal sulcus (PIT and AIT). Unpaired *t* tests (*p* = 0.05 Bonferroni corrected) were then applied to each ROI using data from individual runs of muscimol and saline sessions.

### ROI-Based Analysis: Group Data

Regions of interest were functionally defined based on the clusters of contiguous voxels that were significantly (FWE, *p* < 0.05) activated in the contrast [CS–CC]–[FS–FC] (in which CS = curved stereo, CC = curved control, FS = flat stereo, FC = flat control) in the group data (four monkeys) of the standard fMRI experiment (without saline or muscimol). Based on these data, we defined four ROIs for which we wanted to assess the effect of reversible inactivation of area CIP: two regions in the intraparietal sulcus (anterior IPS and CIP/PIP) and two regions in the inferior temporal cortex (PIT and AIT). The functionally-defined ROI of PIT was located on the shoulder of the lower bank of the STS, largely corresponding to OTd (average within a sphere of 2 mm diameter [20]). The functionally-defined ROI of AIT was located more anteriorly in area TE, the most anterior part of the ITC. Our AIT ROI overlapped somewhat with PITv of [26] in its most caudal portion, but because the activations were mostly located in area TE, on the temporal convexity and more anteriorly in the lower bank of the STS, we considered all activated voxels in this ROI as belonging to AIT. We calculated the interaction effect between the factors curvature (curved versus flat) and stereo (disparity versus control) on the percent signal changes within all activated voxels of the saline sessions in these ROIs with the MarsBaR ROI toolbox for SPM, for muscimol sessions and saline control sessions independently. We then performed two-tailed *t* tests (*p* = 0.05 Bonferroni corrected for multiple comparisons) on the data of individual runs to evaluate the effect of CIP inactivation on our four ROIs. In these same four ROIs, we also calculated the main effect of disparity using the contrast (curved stereo + flat stereo)–(curved control + flat control), and used two-tailed *t* tests across runs to assess the effect of CIP inactivation. Anatomical ROIs (V1v, V1d, V2v, V2d, V3v, V3d, V3a, V4v, V4d, 45a, 45b, F5a, F5p, FST, LST, MSTd, MSTv, MT, UB1, UB2, STP) were defined on a Caret Atlas based on previous studies [26].

## Supporting Information

S1 Fig Methods. A. Stimuli used in the fMRI-inactivation experiments. We used a 2x2 design with factors curvature (curved or flat) and disparity (stereo or control). Above the anaglyphs are icons illustrating the depth structures of the stimuli (red dot represents the fixation point). The control stimuli were the monocular images of the stereo stimuli presented to both eyes simultaneously, so that the visual inputs were matched between the stereo and the control conditions. B. Coronal (left), sagittal (right), and horizontal (bottom) anatomical MRI sections illustrating the injection of 4 **μ**l of a 2% Dotarem (Guerbet, France) solution into the lateral bank of the caudal IPS in monkey S.
(TIF)

S2 Fig Effect of CIP inactivation on percent signal change (PSC) in two paths. A. CIP-inactivation effect on PSC (curvature x disparity interaction effect) on a path drawn along the IPS (left panel) and along the ITC (right panel). Green: saline sessions; red: muscimol sessions. * = *p* < 0.05 uncorrected, ** = *p* < 0.01 uncorrected. Injection syringe indicates inactivated area. B. CIP-inactivation effect on PSC in the main effect of disparity (all disparity conditions–all control conditions) on a path drawn along the IPS (left panel) and the ITC (right panel). Same conventions as in A. Green and red lines indicate SEM. Raw data in [27], int_group_ips.xls, int_group_itc.xls, main_group_ips.xls, main_group_itc.xls.
(TIF)

S3 Fig A. Depth structure activations in the anterior IPS in individual monkeys plotted on their average EPIs during saline (left panels) and muscimol (right panels) sessions. B. Depth structure activations in the ITC in individual monkeys plotted on their average EPIs during saline (left panels) and muscimol (right panels) sessions. C. Coronal sections displaying the *t* values for the depth structure activations during saline minus the *t* values for the depth structure activations during muscimol (group average, *p* < 0.05, FWE corrected).
(TIF)

S4 Fig Individual data. Effect of CIP inactivation on PSC of the curvature x disparity interaction effect in eight functionally-defined ROIs (in both hemispheres) in three animals (upper panel: monkey M, middle panel: monkey S, lower panel: monkey R). Green bars: saline sessions; red bars: muscimol sessions. * = *p* < 0.05, ** = *p* < 0.01. Black lines indicate SEM over runs. Raw data in PSCMonkey_sal_area.xls and PSCMonkey_mus_area.xls in [27].
(TIF)

S5 Fig Percent signal change for individual sessions. PSC for the contrast [CS-CC]-[FS-FC] was calculated in the functionally defined ROIs aIPS, cIPS, and AIT for each individual no-injection, saline, and muscimol session. The PSC for each muscimol session was compared with the average over all saline sessions in the same animal to check for significance; *p* <0.05 indicated by *. Horizontal dashed lines indicate mean PSC per condition. Raw data in [27], PSCMonkey_sal/musc_area_data.xls.
(TIF)

S6 Fig Effect of CIP inactivation in the right hemisphere on the main effect of disparity. A. Flat map showing the fMRI activations in the main effect of stereo (all stereo conditions–all control conditions). Blue outlines: saline sessions; hot colors: muscimol sessions. B. PSC in the main effect of stereo in saline sessions (green bars) and in muscimol sessions (red bars), in the left (left panel) and right (right panel) hemispheres. * = *p* < 0.05, ** = *p* < 0.01. Raw values in [27], PSC_main_group.
(TIF)

S7 Fig Effect of CIP inactivation on behavior. A. Percent concave responses was plotted as a function of the percent coherence, for control (red) and muscimol (blue) sessions. Behavioral data were fitted with a logistic function. Raw values in [27], monkey_percentcorrect.xls. B. Average horizontal eye position signal (right eye only) for convex (red) and concave (blue) choices, for monkey R (left panel) and monkey S (right panel) in sessions without CIP inactivation. The vertical red line indicates stimulus onset, the vertical black line indicates the time point at which the two eye traces start to diverge (based on ROC analysis, *p* < 0.05). C. Average horizontal eye position signals (right eye only) for convex (red) and concave (blue) choices, for monkey R (left panel) and monkey S (right panel) in sessions with CIP inactivation. In both monkeys, the time point at which the two eye traces diverge occurs later (by 10 ms in monkey R and by 20 ms in monkey S) during muscimol sessions. Same conventions as in B. Average of 12,007 and 9,965 trials for monkeys R and S, respectively. Raw data in [27], monkey_eye_mus and monkey_eye_sal.
(TIF)

S8 Fig EM-fMRI results. A. Anatomical MRIs showing electrode locations in CIP for the two animals used (monkey M and monkey D). Below the MRIs are peristimulus-time histograms (PSTHs) of a CIP neuron (recorded at that location), which responded selectively to a planar tilted surface (top part inclined toward the observer) at different positions in depth (columns), indicating higher-order disparity selectivity. Raw data in [27], monkeyD_CIP_date.xls. B. Anatomical MRIs showing locations of electrodes in PIP in the three animals used (monkeys D, R, and M). Below the MRIs are PSTHs of a PIP neuron (recorded at that location) responding selectively to a planar tilted surface (bottom part inclined toward the observer) at different positions in depth, indicating higher-order disparity selectivity. Raw data in [27], monkeyD_PIP_date.xls. C. Individual CIP- (left panels) and PIP-EM (right panels) results for monkeys D, M, and R plotted on the M12 template (at *p* < 0.001 uncorrected).
(TIF)

S9 Fig Effect of CIP inactivation on PSC in the IPS. The bar graph (top panel) shows the PSC of the curvature x disparity interaction effect calculated on consecutive coronal slices from posterior (caudal IPS) to anterior (anterior IPS ROI). For every coronal slice, we considered all voxels that were significantly activated (at *p* < 0.05 corrected for multiple comparisons) by the curvature x disparity interaction. Blue bars: saline session; red bars: muscimol sessions. * = *p* < 0.05 uncorrected. To indicate the anterior–posterior level, the coronal images below illustrate the fMRI activations (curvature x disparity interaction effect) at three levels (caudal IPS, mid IPS, and anterior IPS). Raw data in [27], PSC_S9_group_IPS.
(TIF)

## Abbreviations

AIP: anterior intraparietal area
AIT: anterior inferotemporal cortex
CC: curved control
CIP: caudal intraparietal area
CS: curved stereo
EM: electrical microstimulation
EPI: echo planar image
FC: flat control
fMRI: functional magnetic resonance imaging
FS: flat stereo
FWE: family wise error
IPS: intraparietal sulcus
ITC: inferotemporal cortex
LOC: lateral occipital complex
PIP: the posterior intraparietal area
PIT: posterior inferotemporal cortex
PMTS: posterior middle temporal sulcus
PSC: percent signal change
ROI: region of interest
STS: superior temporal sulcus
TEO: posterior area in inferotemporal cortex
TE: anterior area in inferotemporal cortex
