# Supplementary figures and images for: Posterior Parietal Cortex Drives Inferotemporal Activations During Three-Dimensional Object Vision
Source: PLoS Biol. 2016 Apr 15;14(4):e1002445. doi: 10.1371/journal.pbio.1002445 (PMC4833303; doi:10.1371/journal.pbio.1002445)

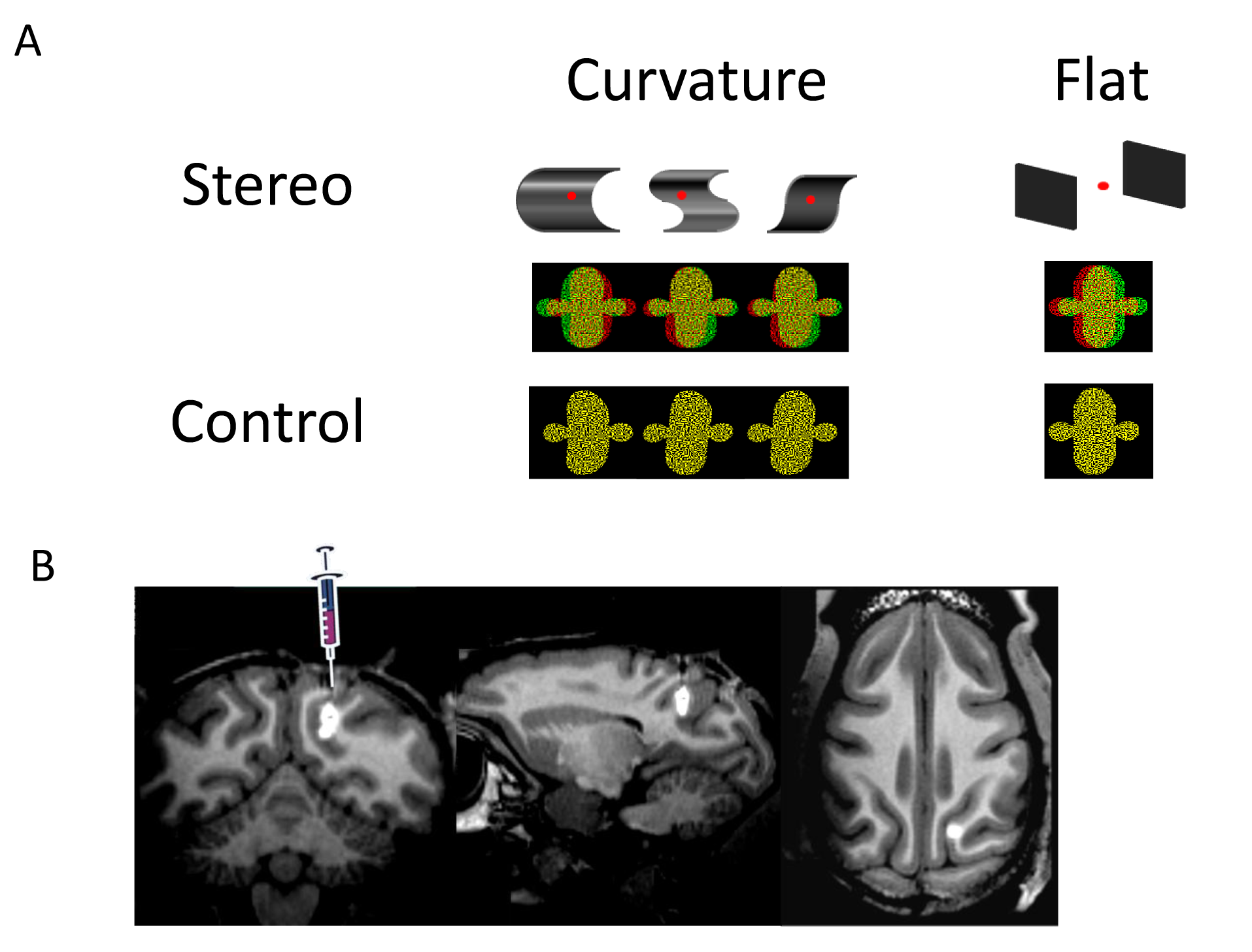

Supplement: S1 Fig — A. Stimuli used in the fMRI-inactivation experiments. We used a 2x2 design with factors curvature (curved or flat) and disparity (stereo or control). Above the anaglyphs are icons illustrating the depth structures of the stimuli (red dot represents the fixation point). The control stimuli were the monocular images of the stereo stimuli presented to both eyes simultaneously, so that the visual inputs were matched between the stereo and the control conditions. B. Coronal (left), sagittal (right), and horizontal (bottom) anatomical MRI sections illustrating the injection of 4 μl of a 2% Dotarem (Guerbet, France) solution into the lateral bank of the caudal IPS in monkey S. (TIF) [file pbio.1002445.s001.tif]

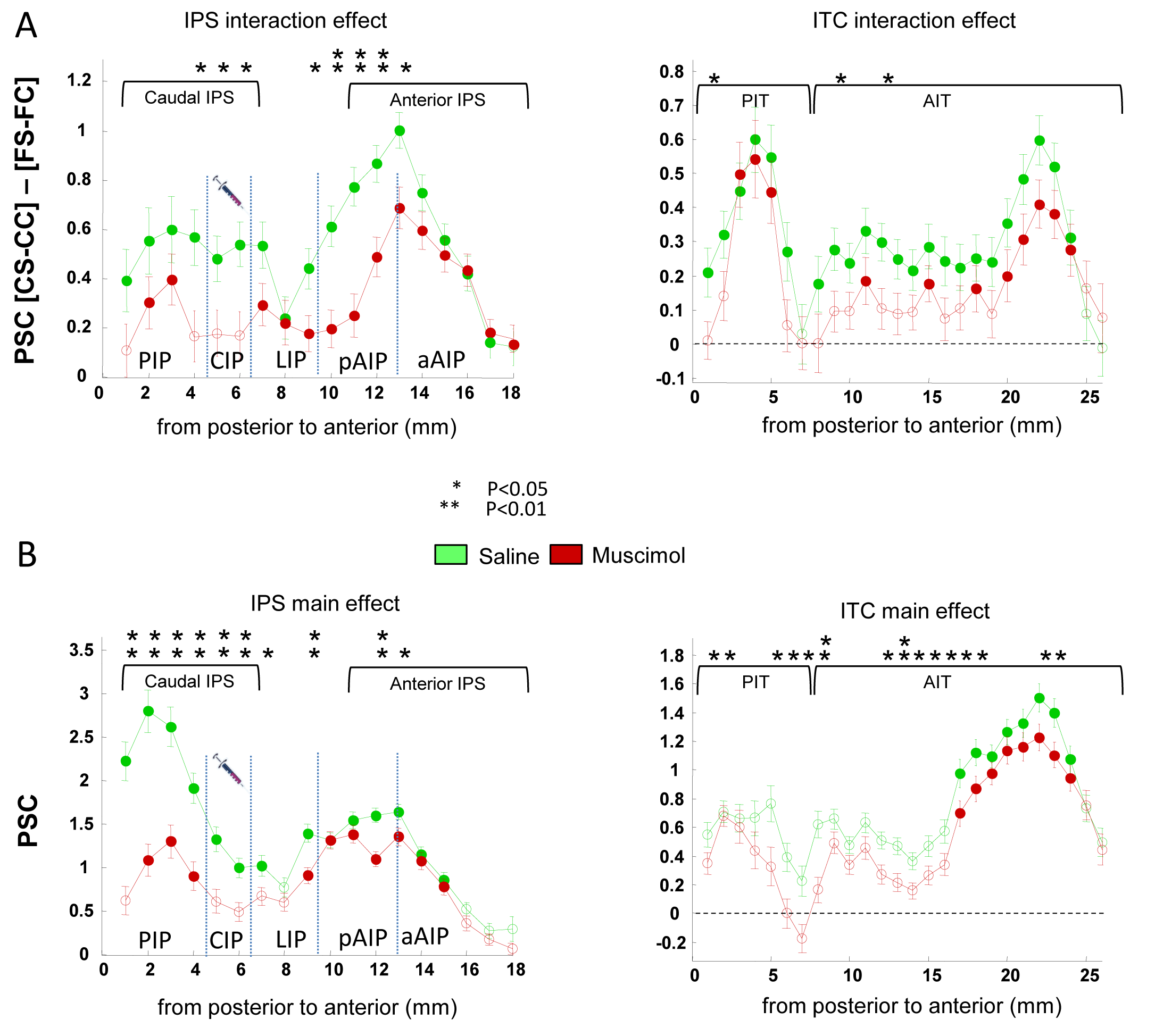

Supplement: S2 Fig — A. CIP-inactivation effect on PSC (curvature x disparity interaction effect) on a path drawn along the IPS (left panel) and along the ITC (right panel). Green: saline sessions; red: muscimol sessions. * = p < 0.05 uncorrected, ** = p < 0.01 uncorrected. Injection syringe indicates inactivated area. B. CIP-inactivation effect on PSC in the main effect of disparity (all disparity conditions–all control conditions) on a path drawn along the IPS (left panel) and the ITC (right panel). Same conventions as in A. Green and red lines indicate SEM. Raw data in [27], int_group_ips.xls, int_group_itc.xls, main_group_ips.xls, main_group_itc.xls. (TIF) [file pbio.1002445.s002.tif]

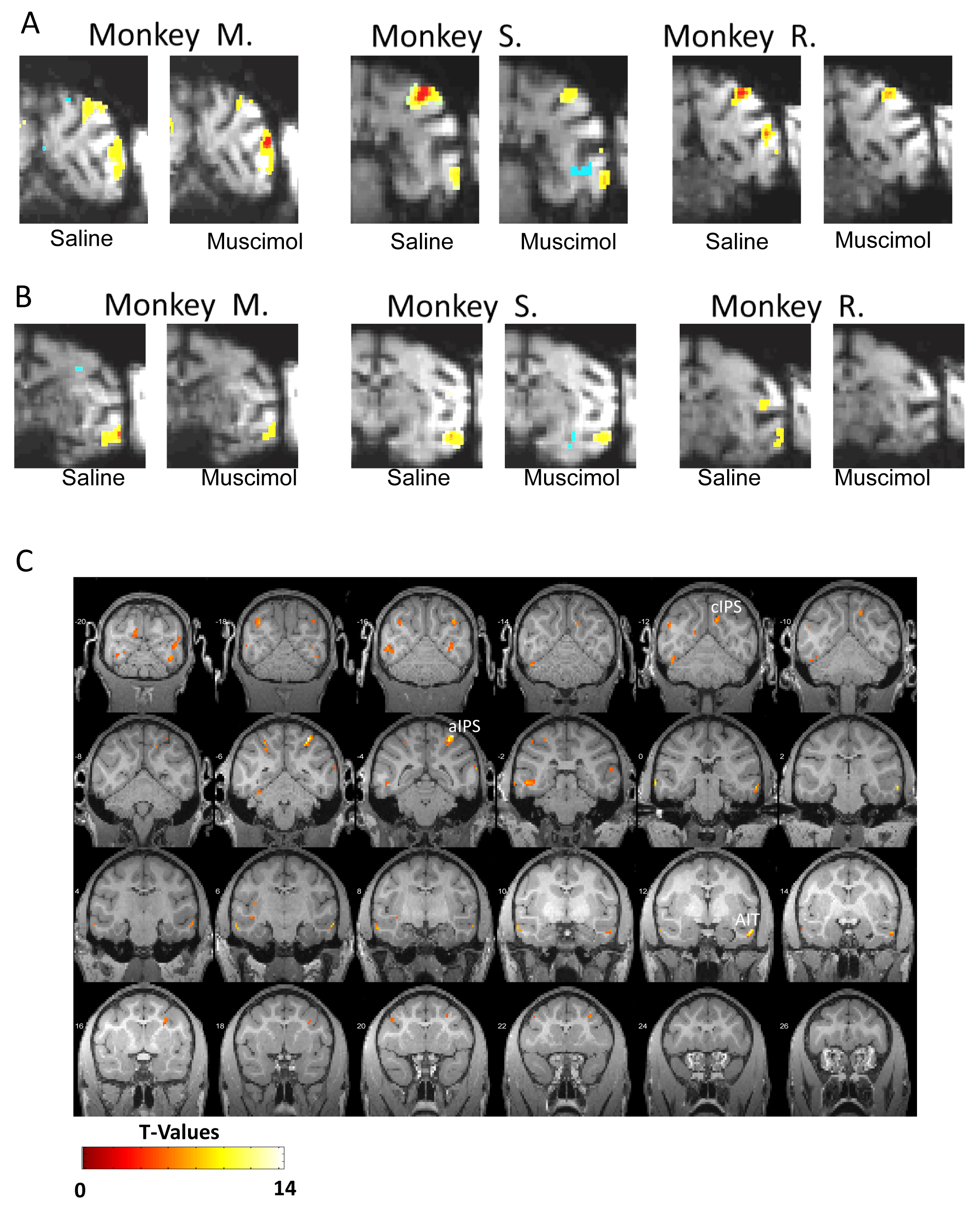

Supplement: S3 Fig — A. Depth structure activations in the anterior IPS in individual monkeys plotted on their average EPIs during saline (left panels) and muscimol (right panels) sessions. B. Depth structure activations in the ITC in individual monkeys plotted on their average EPIs during saline (left panels) and muscimol (right panels) sessions. C. Coronal sections displaying the t values for the depth structure activations during saline minus the t values for the depth structure activations during muscimol (group average, p < 0.05, FWE corrected). (TIF) [file pbio.1002445.s003.tif]

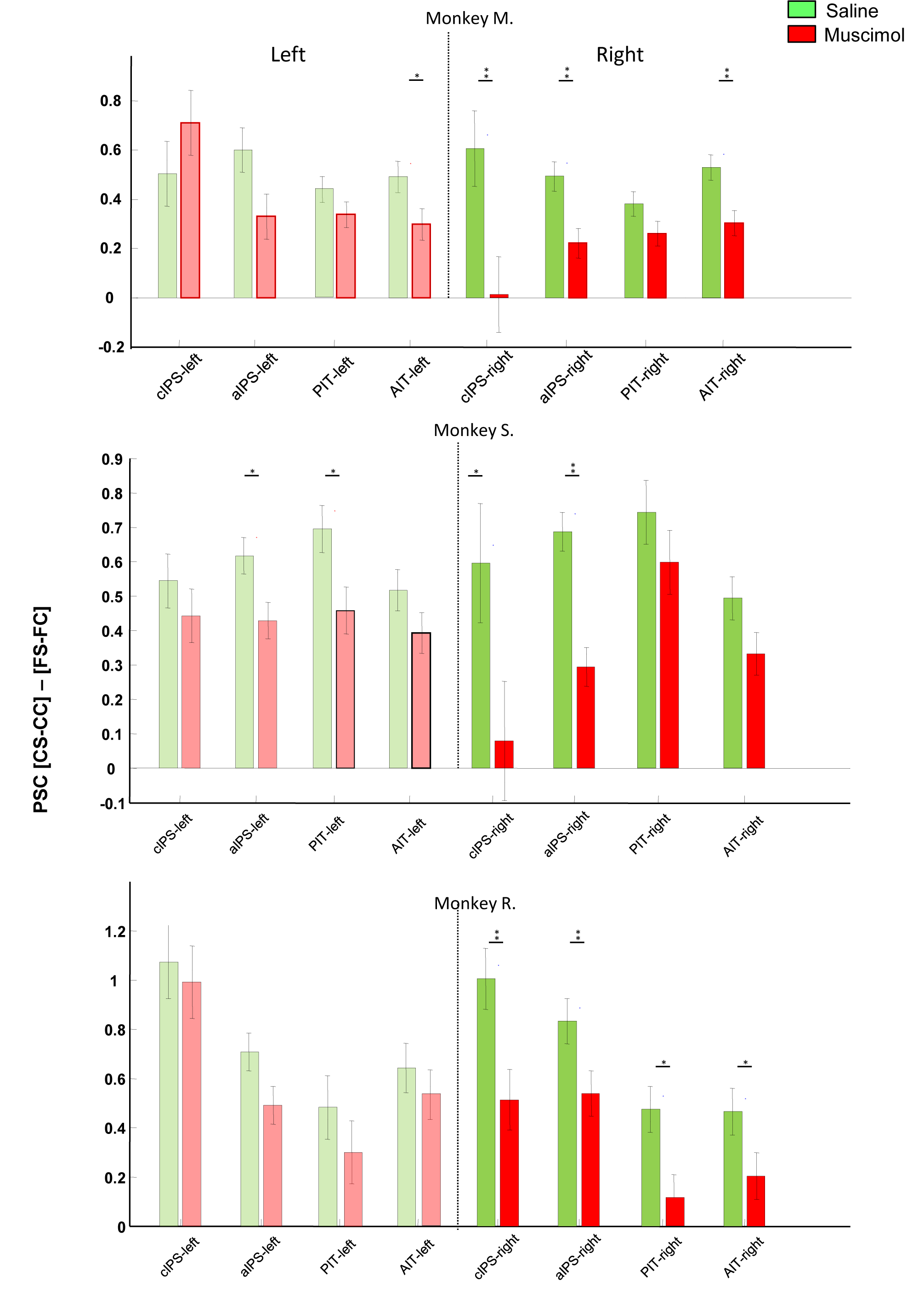

Supplement: S4 Fig — Effect of CIP inactivation on PSC of the curvature x disparity interaction effect in eight functionally-defined ROIs (in both hemispheres) in three animals (upper panel: monkey M, middle panel: monkey S, lower panel: monkey R). Green bars: saline sessions; red bars: muscimol sessions. * = p < 0.05, ** = p < 0.01. Black lines indicate SEM over runs. Raw data in PSCMonkey_sal_area.xls and PSCMonkey_mus_area.xls in [27]. (TIF) [file pbio.1002445.s004.tif]

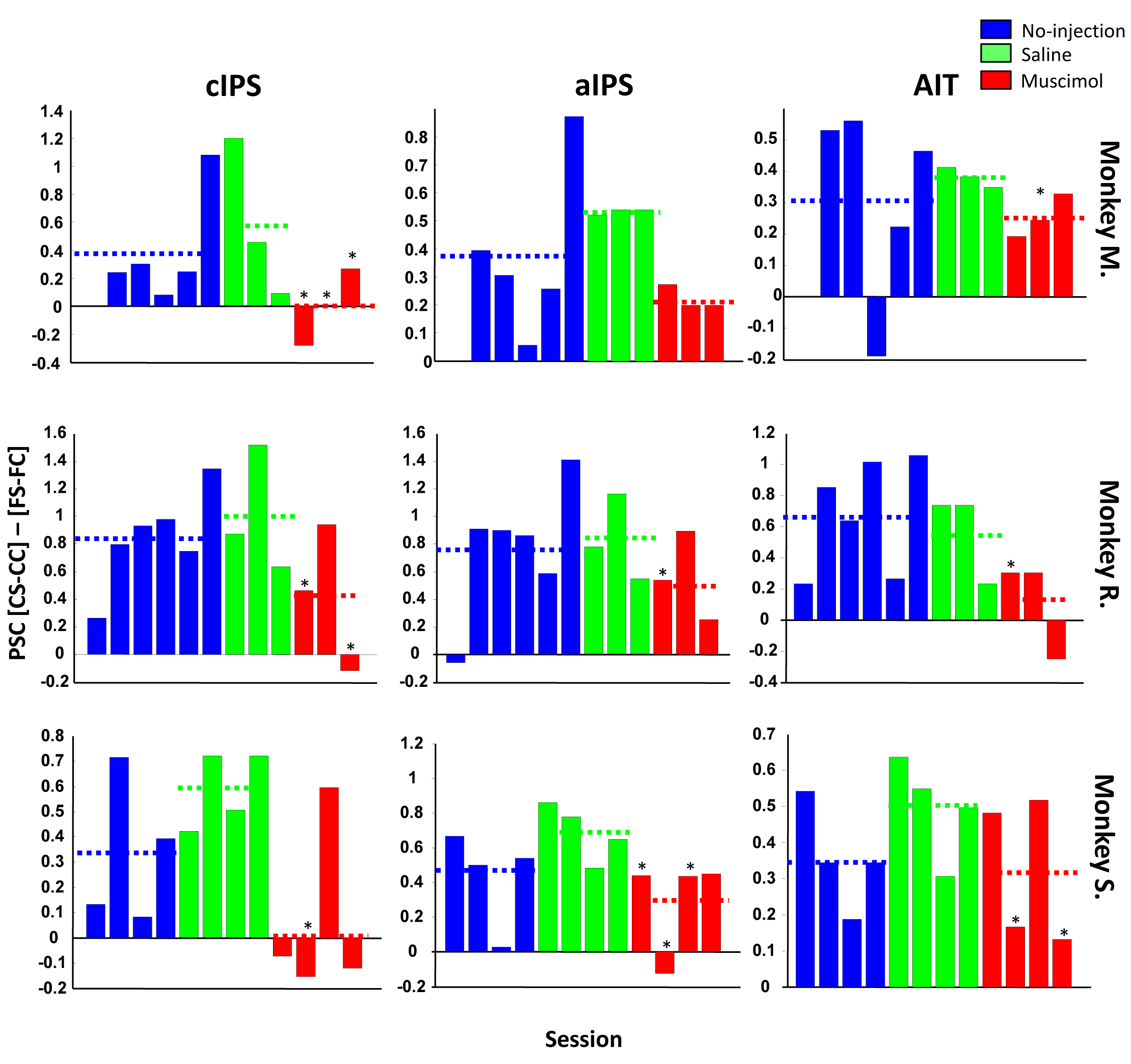

Supplement: S5 Fig — PSC for the contrast [CS-CC]-[FS-FC] was calculated in the functionally defined ROIs aIPS, cIPS, and AIT for each individual no-injection, saline, and muscimol session. The PSC for each muscimol session was compared with the average over all saline sessions in the same animal to check for significance; p <0.05 indicated by *. Horizontal dashed lines indicate mean PSC per condition. Raw data in [27], PSCMonkey_sal/musc_area_data.xls. (TIF) [file pbio.1002445.s005.tif]

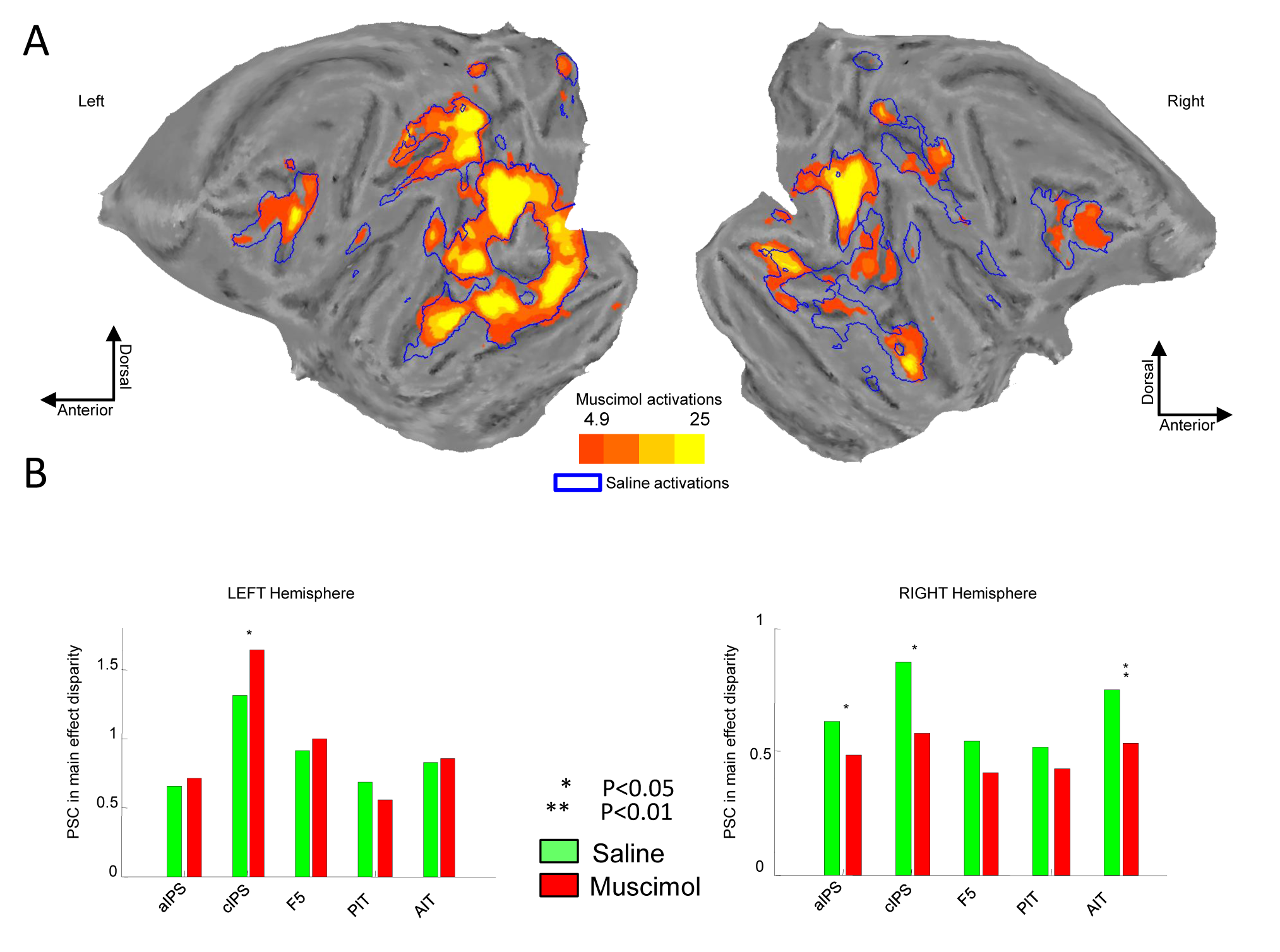

Supplement: S6 Fig — A. Flat map showing the fMRI activations in the main effect of stereo (all stereo conditions–all control conditions). Blue outlines: saline sessions; hot colors: muscimol sessions. B. PSC in the main effect of stereo in saline sessions (green bars) and in muscimol sessions (red bars), in the left (left panel) and right (right panel) hemispheres. * = p < 0.05, ** = p < 0.01. Raw values in [27], PSC_main_group. (TIF) [file pbio.1002445.s006.tif]

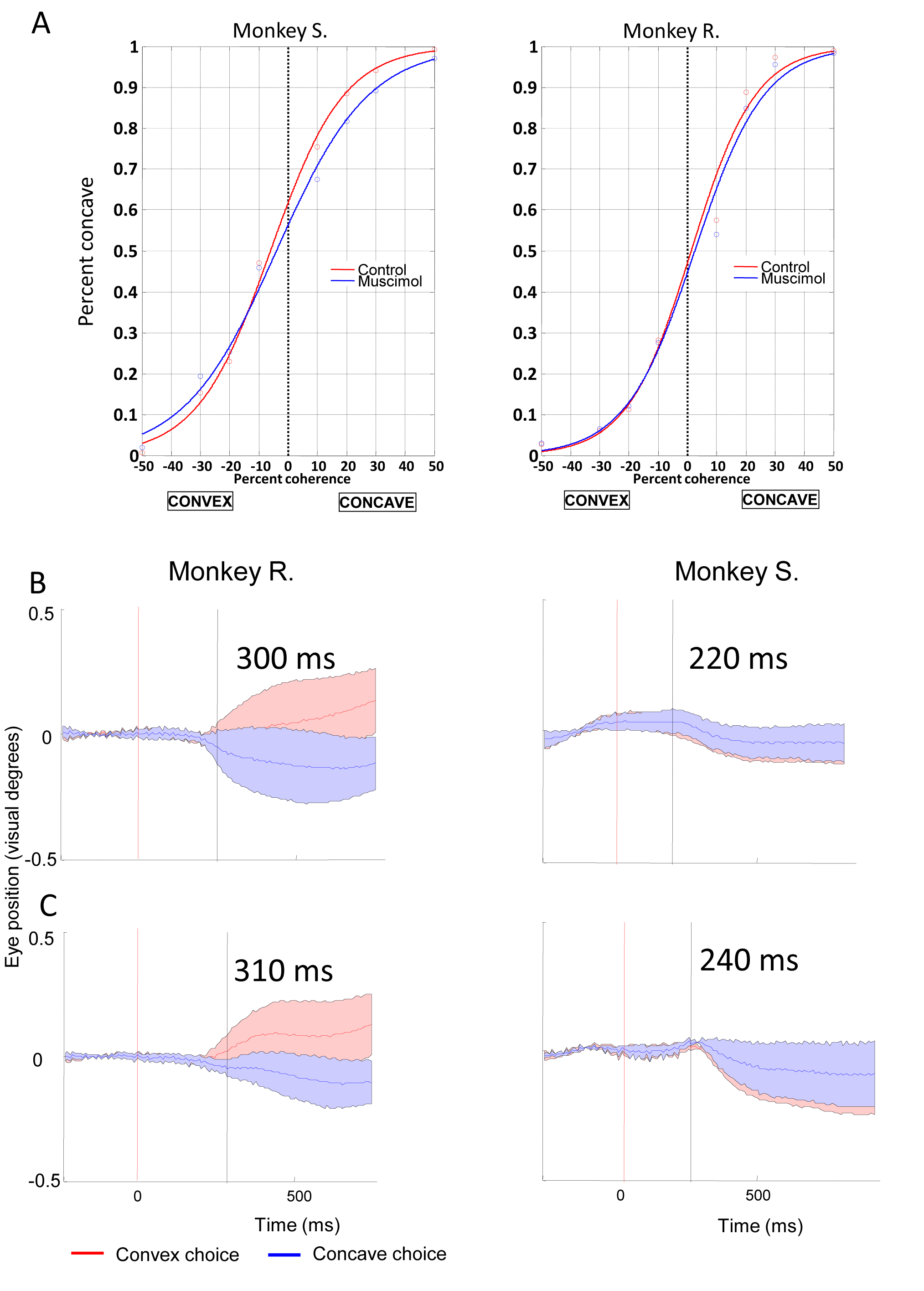

Supplement: S7 Fig — A. Percent concave responses was plotted as a function of the percent coherence, for control (red) and muscimol (blue) sessions. Behavioral data were fitted with a logistic function. Raw values in [27], monkey_percentcorrect.xls. B. Average horizontal eye position signal (right eye only) for convex (red) and concave (blue) choices, for monkey R (left panel) and monkey S (right panel) in sessions without CIP inactivation. The vertical red line indicates stimulus onset, the vertical black line indicates the time point at which the two eye traces start to diverge (based on ROC analysis, p < 0.05). C. Average horizontal eye position signals (right eye only) for convex (red) and concave (blue) choices, for monkey R (left panel) and monkey S (right panel) in sessions with CIP inactivation. In both monkeys, the time point at which the two eye traces diverge occurs later (by 10 ms in monkey R and by 20 ms in monkey S) during muscimol sessions. Same conventions as in B. Average of 12,007 and 9,965 trials for monkeys R and S, respectively. Raw data in [27], monkey_eye_mus and monkey_eye_sal. (TIF) [file pbio.1002445.s007.tif]

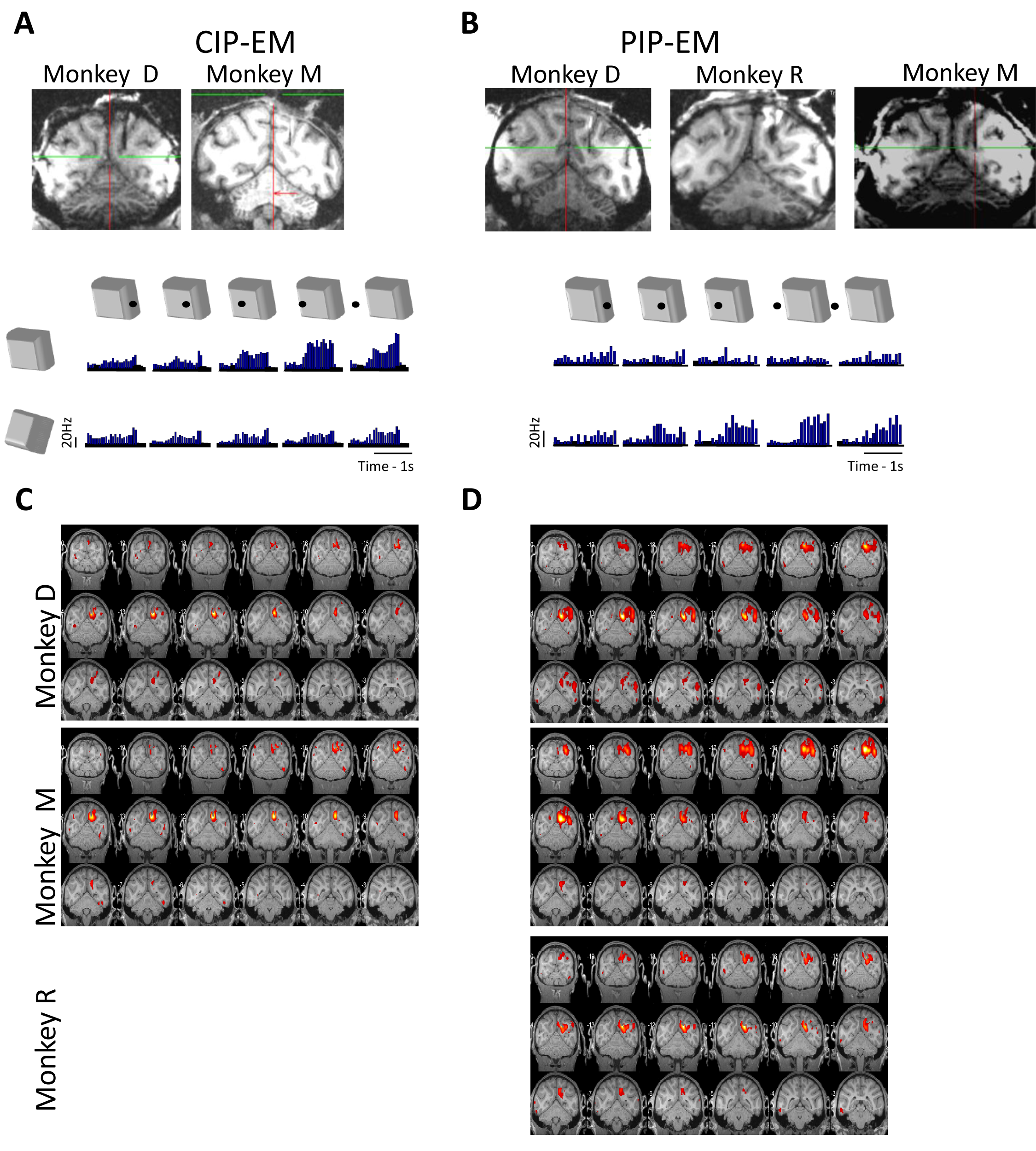

Supplement: S8 Fig — A. Anatomical MRIs showing electrode locations in CIP for the two animals used (monkey M and monkey D). Below the MRIs are peristimulus-time histograms (PSTHs) of a CIP neuron (recorded at that location), which responded selectively to a planar tilted surface (top part inclined toward the observer) at different positions in depth (columns), indicating higher-order disparity selectivity. Raw data in [27], monkeyD_CIP_date.xls. B. Anatomical MRIs showing locations of electrodes in PIP in the three animals used (monkeys D, R, and M). Below the MRIs are PSTHs of a PIP neuron (recorded at that location) responding selectively to a planar tilted surface (bottom part inclined toward the observer) at different positions in depth, indicating higher-order disparity selectivity. Raw data in [27], monkeyD_PIP_date.xls. C. Individual CIP- (left panels) and PIP-EM (right panels) results for monkeys D, M, and R plotted on the M12 template (at p < 0.001 uncorrected). (TIF) [file pbio.1002445.s008.tif]

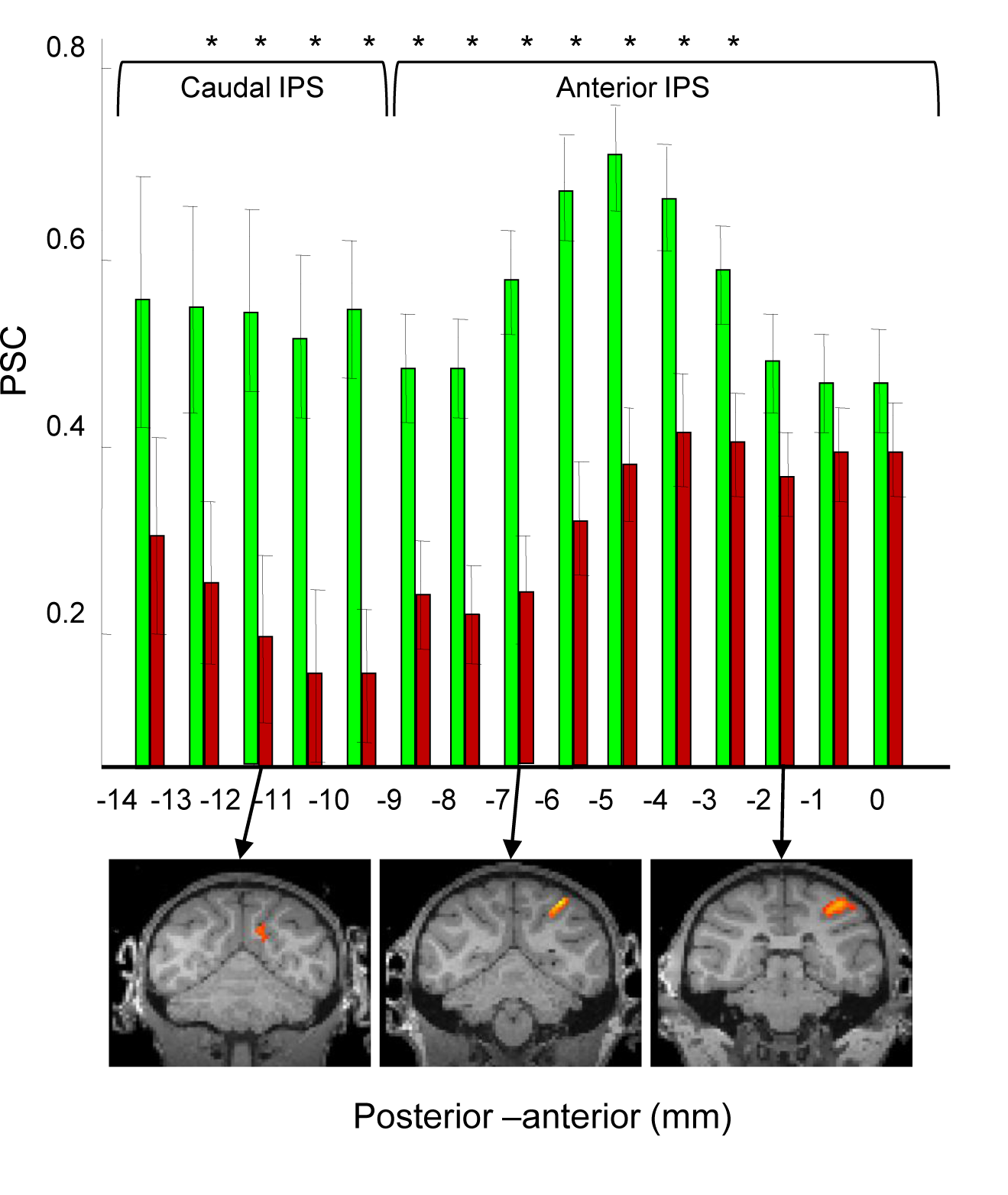

Supplement: S9 Fig — The bar graph (top panel) shows the PSC of the curvature x disparity interaction effect calculated on consecutive coronal slices from posterior (caudal IPS) to anterior (anterior IPS ROI). For every coronal slice, we considered all voxels that were significantly activated (at p < 0.05 corrected for multiple comparisons) by the curvature x disparity interaction. Blue bars: saline session; red bars: muscimol sessions. * = p < 0.05 uncorrected. To indicate the anterior–posterior level, the coronal images below illustrate the fMRI activations (curvature x disparity interaction effect) at three levels (caudal IPS, mid IPS, and anterior IPS). Raw data in [27], PSC_S9_group_IPS. (TIF) [file pbio.1002445.s009.tif]
